# Presynaptic cGMP sets synaptic strength in the striatum and is important for motor learning

**DOI:** 10.15252/embr.202154361

**Published:** 2022-06-23

**Authors:** Tim Fieblinger, Alberto Perez‐Alvarez, Paul J Lamothe‐Molina, Christine E Gee, Thomas G Oertner

**Affiliations:** ^1^ Institute for Synaptic Physiology University Medical Center Hamburg‐Eppendorf Hamburg Germany; ^2^ Rapp OptoElectronic GmbH Wedel Germany

**Keywords:** striatum, synapse, transmitter release, gain control, cyclic nucleotides, Neuroscience

## Abstract

The striatum is a subcortical brain region responsible for the initiation and termination of voluntary movements. Striatal spiny projection neurons receive major excitatory synaptic input from neocortex and thalamus, and cyclic nucleotides have long been known to play important roles in striatal function. Yet, the precise mechanism of action is unclear. Here, we combine optogenetic stimulation, 2‐photon imaging, and genetically encoded scavengers to dissect the regulation of striatal synapses in mice. Our data show that excitatory striatal inputs are tonically depressed by phosphodiesterases (PDEs), in particular PDE1. Blocking PDE activity boosts presynaptic calcium entry and glutamate release, leading to strongly increased synaptic transmission. Although PDE1 degrades both cAMP and cGMP, we uncover that the concentration of cGMP, not cAMP, controls the gain of striatal inputs. Disturbing this gain control mechanism *in vivo* impairs motor skill learning in mice. The tight dependence of striatal excitatory synapses on PDE1 and cGMP offers a new perspective on the molecular mechanisms regulating striatal activity.

## Graphical abstract

The strength of striatal input synapses is controlled by the activity of phosphodiesterases and cGMP. Disturbing this gain control mechanism in corticostriatal synapses impairs motor learning in mice.

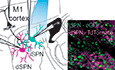

## Introduction

The striatum is the primary entry point to the basal ganglia. A vast number of excitatory inputs from various cortical and thalamic areas converge onto the dendrites of striatal spiny projection neurons (SPNs). Integration and adaptation of these inputs are critical for shaping SPN output activity, appropriate movement control, and motor learning (Costa, 2007; Klaus *et al*, 2019). Conversely, dysfunction of striatal synapses and SPN output activity is a leading cause of motor symptoms in dystonia, Parkinson's and Huntington's disease. Cyclic nucleotides such as cyclic adenosine monophosphate (cAMP) and cyclic guanosine monophosphate (cGMP) are important second messengers in neurons. In the striatum, cAMP signaling is fundamental to principal neuron function, and its disturbance plays a critical role in various diseases and their therapies (Nairn *et al*, 2004; Goto, 2017; Heckman *et al*, 2018; Koch & Raymond, 2019). While the importance of cAMP for synaptic transmission and plasticity is well established (Nicoll & Schmitz, 2005; Girault, 2012; Kandel, 2012; Zhai *et al*, 2019), the role of cGMP is less well understood. Both cyclic nucleotides are rapidly hydrolyzed by phosphodiesterases (PDEs) which constrain their signaling in time and space. Phosphodiesterases constitute a superfamily of enzymes, spanning over 11 families, often with multiple genes and splice variants (Keravis & Lugnier, 2012). Many PDEs are found in the striatum at considerably high levels (Menniti *et al*, 2006; Threlfell & West, 2013). Here, we set out to dissect the role of cyclic nucleotides and PDEs in excitatory synaptic transmission onto SPNs of the dorsolateral striatum, combining pharmacology with targeted expression of genetically encoded tools. We show that inhibition of PDEs increases excitatory inputs to both classes of SPNs, exerting parallel control on the *direct* and the *indirect* pathway. We detected constitutively active PDE at both cortico‐ and thalamostriatal synapses, but to our surprise, the effects on glutamate release were not mediated by cAMP. Two lines of evidence, pharmacological inhibition of the cGMP‐dependent protein kinase 1 and expression of a genetically encoded cGMP‐scavenger protein, point to cGMP as key regulator of synaptic strength in the striatum. The importance of this regulatory system was also apparent in behavioral experiments where a genetic block of cGMP signaling in corticostriatal projections impaired motor skill learning in mice.

## Results

### Inhibition of phosphodiesterases increases excitatory synaptic transmission in the striatum

**Figure 1:**
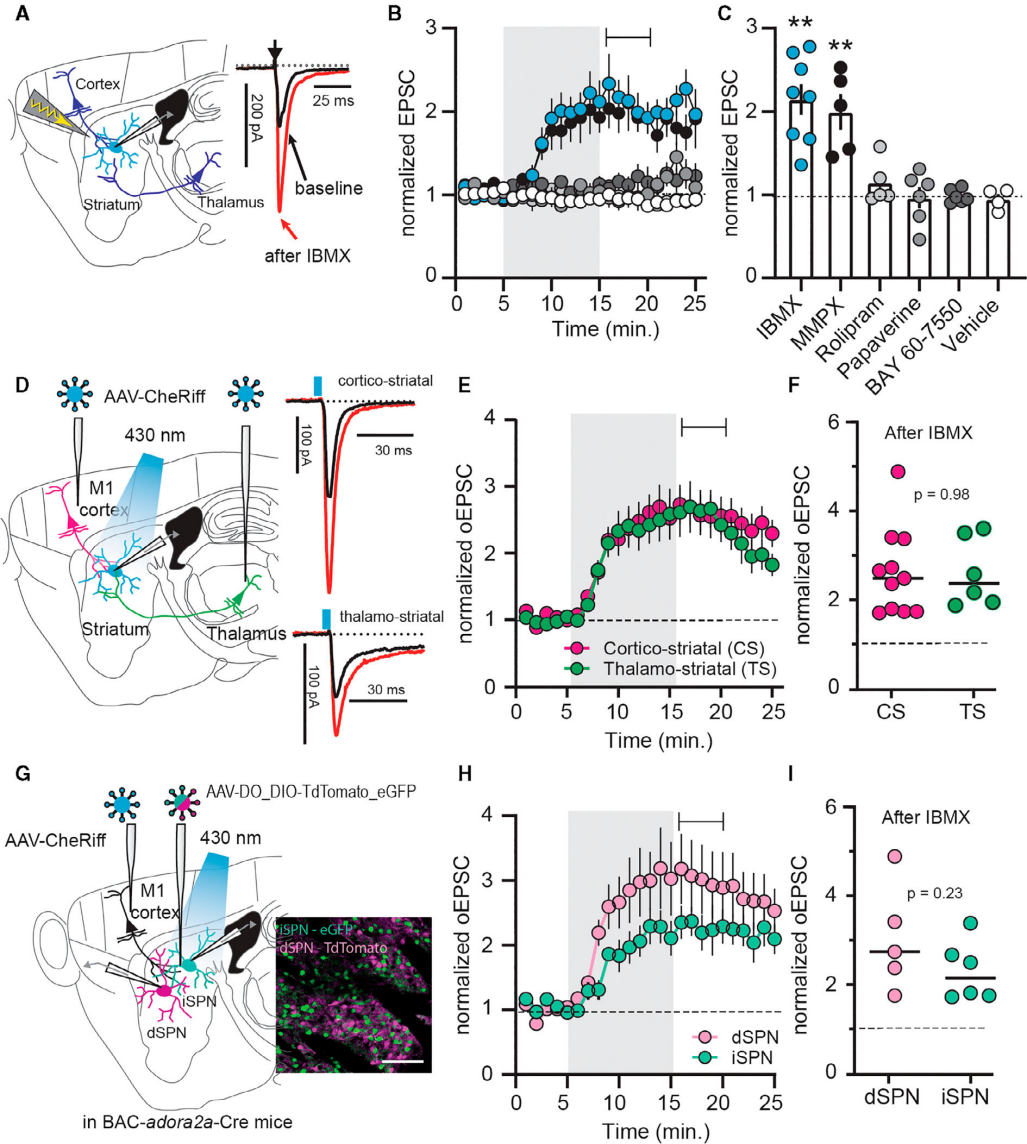
Phosphodiesterase 1 limits the strength of cortico‐ and thalamostriatal synapses AElectrical stimulation of descending afferents evoked excitatory postsynaptic currents (EPSCs) in striatal spiny projection neurons (SPNs). EPSCs before (*black*) and after (*red*) application of the phosphodiesterase (PDE) inhibitor IBMX (75 μM).BNormalized EPSC recordings. Shaded area indicates washin of IBMX, the PDE1 inhibitor MMPX (10 μM), the PDE4 inhibitor rolipram (1 μM), the PDE10 inhibitor papaverine (10 μM), the PDE2 inhibitor BAY 66‐7550 (2 μM) or vehicle (DMSO, 0.002%). Mean ± SEM.CAveraged EPSCs during the 5‐min interval indicated in (B) ** *P* < 0.01, paired *t*‐test versus respective baseline, *N* = 5–8. Mean ± SEM.DCheRiff was expressed in either cortical area M1 or the thalamic parafascicular nucleus (one area injected per mouse) and blue light flashes were used to evoke cortical or thalamic EPSCs. Example traces show response before (*black*) and after IBMX (*red*).EOptically evoked EPSCs (oEPSCs) from cortical (*magenta*) or thalamic inputs (*green*). Shaded area is washin of IBMX. Mean ± SEM.FAveraged normalized EPSCs during the interval indicated in (E). *P* = 0.98, unpaired *t*‐test, *N* = 11 and 6.GCortical oEPSCs recorded from GFP‐labeled, Cre‐positive indirect pathway (iSPNs), and tdTomato‐labeled Cre‐negative direct pathway SPNs (dSPNs). Scale bar 100 μm.HNormalized oEPSCs in dSPNs and iSPNs. Shaded area indicates washin of IBMX. Mean ± SEM.IAveraged responses during the interval marked in (H). *P* = 0.23, unpaired *t*‐test, dSPNs versus iSPNs *N* = 5 and 6.

In acute slices of mouse dorsolateral striatum, we performed patch clamp recordings from SPNs, evoking excitatory postsynaptic currents (EPSCs) by local electrical stimulation. The broad‐spectrum PDE‐inhibitor IBMX rapidly increased EPSCs, an effect that persisted even after washout (Fig 1A–C). Thus, under baseline conditions, excitatory synapses were strongly inhibited by PDEs. This augmentation of EPSCs also occurred in the absence of synaptic stimulation (Appendix Fig S1A–C). Several PDEs with distinct pharmacological profiles are expressed in the striatum. We found that inhibition of PDE1, but not inhibition of PDE2, PDE4, or PDE10, mimicked the effect of IBMX (Fig 1B and C).

The stimulation electrode was positioned to preferentially activate descending cortical inputs, but may also have activated thalamic axons. To improve specificity, we stimulated axons originating in primary motor cortex (M1) or the parafascicular nucleus of the thalamus (PF; Fig 1D) with the channelrhodopsin CheRiff. M1 and PF are the two main sources of glutamatergic innervation to the striatum and EPSCs from both inputs increased equally after inhibition of PDEs (Fig 1D–F). Striatal SPNs further divide into two major populations, forming the so‐called *direct* and *indirect* pathways (dSPNs and iSPNs, respectively), which have distinct transcriptional, molecular, electrophysiological, and morphological characteristics (Gertler *et al*, 2008; Heiman *et al*, 2008; Planert *et al*, 2013; Fieblinger *et al*, 2014; Gokce *et al*, 2016; Saunders *et al*, 2018; Fieblinger, 2021). Using viral transduction in a transgenic mouse that expresses Cre‐recombinase selectively in iSPNs (BAC‐*adora2a*‐Cre), Cre‐positive iSPNs were labeled with GFP and Cre‐negative neurons with tdTomato (Fig 1G). Among the latter, dSPNs were identified by their typical electrophysiological profile that distinguishes SPNs from interneurons. In accordance with previous reports (Gertler *et al*, 2008; Planert *et al*, 2013; Fieblinger *et al*, 2014), identified iSPNs and dSPNs displayed characteristic differences in somatic excitability (Appendix Fig S2A and B). Interestingly, EPSCs recorded from both *direct* and *indirect* pathway SPNs increased when PDEs were inhibited (Fig 1G–I). This effect was further confirmed in retrogradely labeled dSPNs (Appendix Fig S2C–E).

### Phosphodiesterases limit glutamate release from synaptic terminals

**Figure 2:**
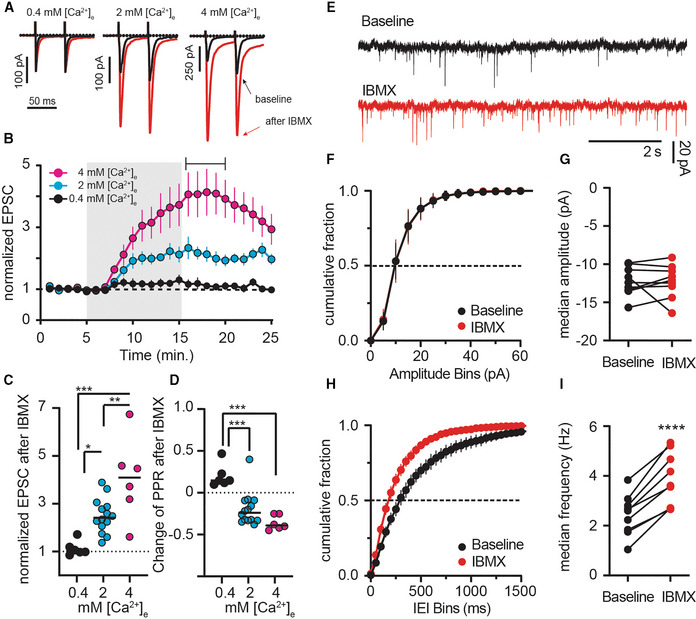
Boosting of synaptic transmission following inhibition of phosphodiesterases is calcium‐dependent APaired‐pulse EPSCs under different extracellular calcium ([Ca^2+^]_e_) conditions pre (*black*) and post IBMX (*red*).BEPSC amplitude in low (0.4 mM, black), normal (2 mM, *cyan*, replotted from Fig 1B), and high (4 mM, *magenta*) [Ca^2+^]_e_. Shading indicates application of IBMX (75 μM). Mean ± SEM.CAverage EPSCs.DPaired‐pulse ratios (PPRs) of individual recordings from time indicated in (B). *P < 0.05, **P < 0.01, ***P < 0.001, ANOVA followed by Tukey's multiple comparisons. *N* = 6–14.EExample mEPSCs recorded in tetrodotoxin before and after IBMX.FCumulative fraction plots of mEPSC amplitude at baseline and after IBMX (75 μM, 10 min). Mean ± SEM.GComparison of the median mEPSC amplitude per cell shows no significant effect of IBMX. paired *t*‐test, *P* = 0.99, *N* = 9.HCumulative fraction plots of mEPSC interevent intervals (IEI) showing a left shift after IBMX. Mean ± SEM.IComparison of median event frequency shows an increase of mEPSC event frequency after IBMX. ****P < 0.0001, paired *t*‐test, *N* = 9.

The absence of specificity for the postsynaptic cell type hints at a presynaptic site of action. Supporting the notion of altered transmitter release, the IBMX‐induced increase in EPSC amplitude was accompanied by a decrease in the paired‐pulse ratio (PPR; Fig 2A and D). Increasing [Ca^2+^]_e_ from 2 to 4 mM enlarged the EPSC boosting effect of IBMX (Fig 2A–C), whereas lowering [Ca^2+^]_e_ to 0.4 mM diminished it and also reversed the IBMX‐induced decrease in PPR (Fig 2A–D). Presynaptic Ca^2+^ influx through voltage‐dependent calcium channels (VDCCs) is required to trigger vesicle release and presynaptic plasticity (Catterall & Few, 2008). Consequently, block of VDCCs by CdCl_2_ almost abolishes synaptic transmission, even after boosting and prevented EPSC potentiation by PDE inhibition (Appendix Fig S3A and B). Consistent with a presynaptic site of action, miniature EPSC (mEPSC) frequency, but not amplitude, increased after PDE inhibition (Fig 2E–I). Taken together, the (i) change in PPR, (ii) dependence on [Ca^2+^]_e_, and (iii) effect on mEPSC frequency suggest that under physiological conditions, synaptic transmitter release is limited by PDEs (Regehr, 2012).

**Figure 3:**
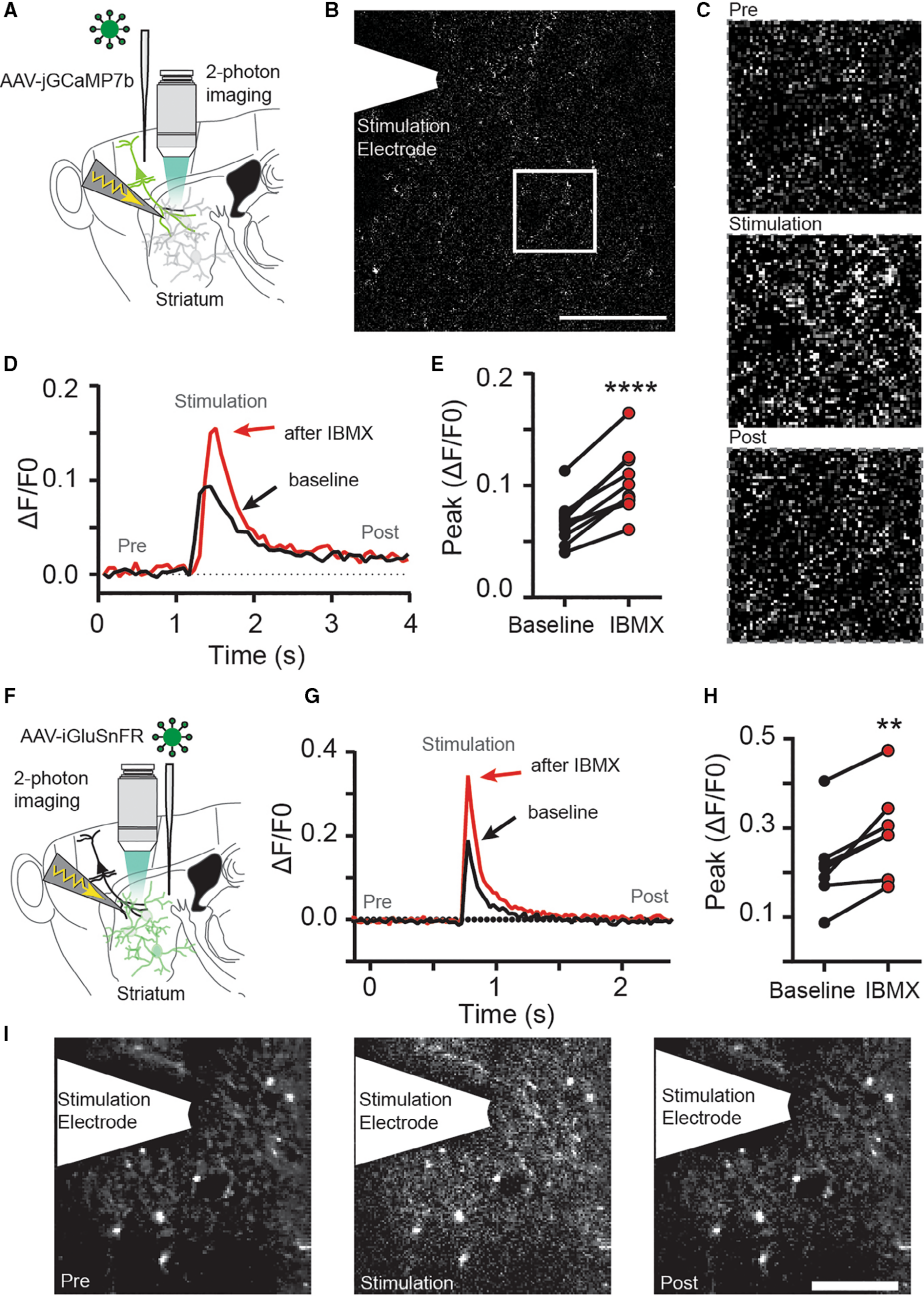
Phosphodiesterases constrain presynaptic glutamate release in the striatum AThe calcium sensor jGCaMP7b was expressed in cortical input neurons to the striatum and electrically evoked signals were imaged in axon terminals in the striatum using fast‐scanning 2‐photon microscopy.BFull‐field image of jGCaMP7b signal in the dorsolateral striatum. Scale bar: 30 μm.CZoom in from (B) and jGCaMP7b signal before (*Pre*), during (*Stimulation*) and after (*Post*) electrical stimulation of afferents.DExample recording of full‐field fluorescence signal, before (*black*) and after IBMX application (*red*).EBlocking phosphodiesterases with IBMX (75 μM, 10 min) increases the peak Ca^2+^ signal detected in presynaptic terminals. ****P < 0.0001, paired *t*‐test. *N* = 11 slices (three mice).FThe glutamate sensor iGluSnFR was expressed in striatal neurons and electrically evoked signals were imaged using fast‐scanning 2‐photon microscopy.GExample of electrically evoked signals before (*black*) and after IBMX (*red*).HPeak fluorescence signal is increased after inhibition of phosphodiesterases with IBMX (75 μM, 10 min). **P < 0.01 paired *t*‐test, *N* = 7 (three mice).IThe striatal iGluSnFR signal before (*Pre*), during (*Stimulation*), and after (*Post*) electrical stimulation of afferents. Scale bar: 25 μm.

To more directly assess whether the effects were presynaptic, we expressed the calcium sensor jGCaMP7b in M1 cortical neurons and imaged evoked presynaptic Ca^2+^‐signals using fast‐scanning 2‐photon microscopy (Fig 3A). As expected, Ca^2+^‐transients were larger after inhibition of PDEs (Fig 3B–E). Likewise, glutamate release in the striatum, as detected by 2‐photon imaging of the genetically encoded glutamate sensor iGluSnFR, increased when PDEs were inhibited (Fig 3F–I). Although the postsynaptic neurons express iGluSnFR, it is indeed the presynaptic release into the synaptic cleft that is being imaged. The iGluSnFR peak fluorescence amplitude increased significantly, as well as the area over which distinct fluorescence signals were detected (Fig 3G and H, Appendix Fig S4A and B). Both the IBMX‐induced increased of evoked presynaptic Ca^2+^ and released glutamate followed a time course similar to the electrophysiological recordings (Appendix Fig S4C). Overall, these results solidify the hypothesis that phosphodiesterase activity limits neurotransmitter release at major excitatory inputs to the striatum by reducing the amplitude of presynaptic Ca^2+^ transients.

### Interaction with mGlu2/3 and GABA_B_ receptor signaling

**Figure 4:**
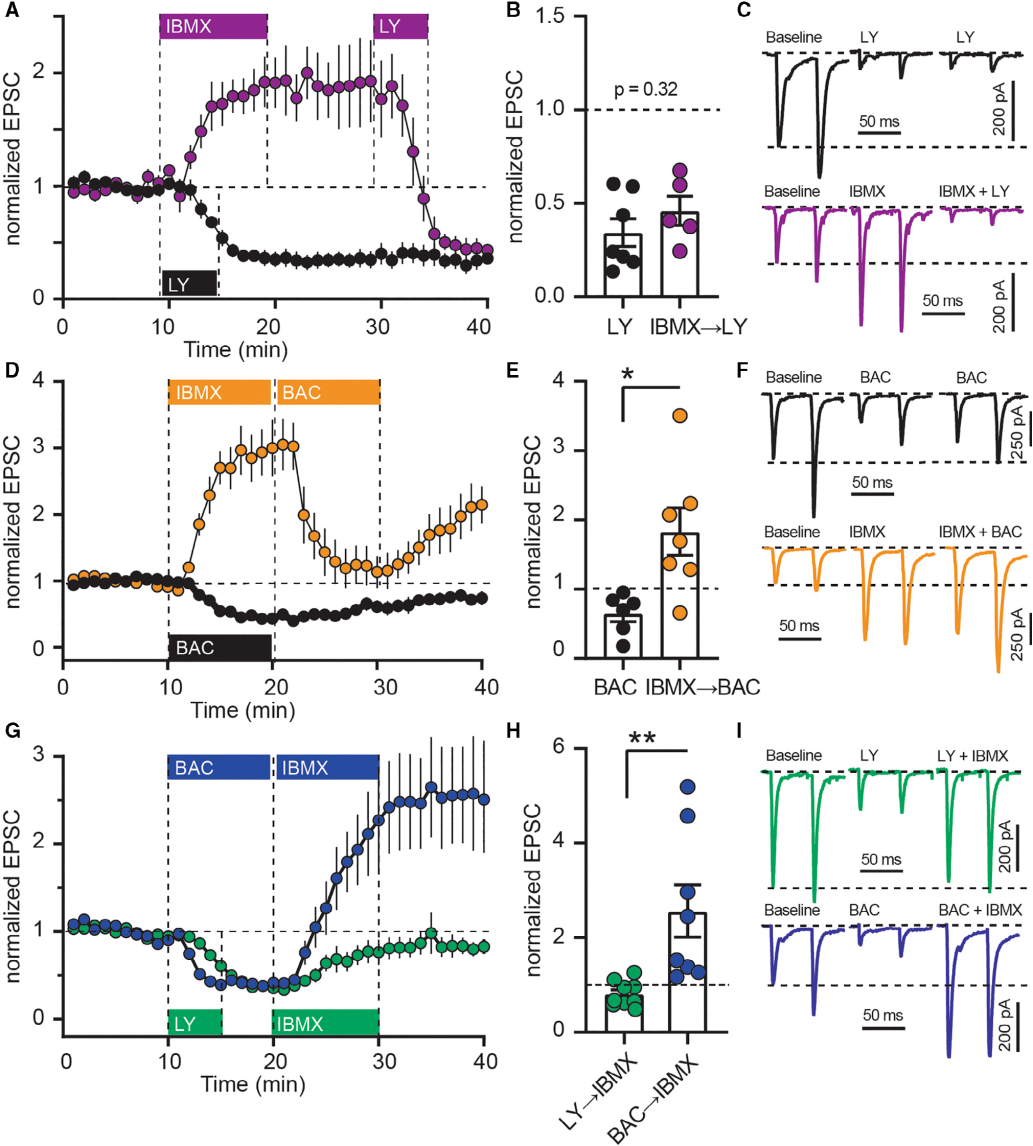
Phosphodiesterase inhibition differentially counteracts synaptic depression induced by mGlu2/3‐ and GABA_B_‐agonists AThe mGlu2/3‐agonist LY379268 (LY; 200 nM) was applied alone (*black*), or after IBMX (75 μM; *purple*). Mean ± SEM.BAveraged EPSC amplitudes from the last 5 min of (A). *P* = 0.32, unpaired *t*‐test, *N* = 5–6. Mean ± SEM.CExample recordings from (A).DGABA_B_ agonist baclofen (BAC; 3 μM) applied alone (*black*), or after IBMX (*orange*). Mean ± SEM.EAveraged EPSC amplitudes from the last 5 min of (D). *P < 0.05, unpaired *t*‐test, *N* = 6–7. Mean ± SEM.FExample recordings from (D).GIMBX was applied after LY (*green*) or BAC (*blue*). Mean ± SEM.HAveraged EPSC amplitudes from the last 5 min of (G). **P < 0.01, unpaired *t*‐test *N* = 6–8. Mean ± SEM.IExample recordings from (G).

Interestingly, reduced calcium entry via VDCCs has been shown to mediate synaptic depression in the striatum, for example, following activation of presynaptic metabotropic glutamate receptors 2/3 (mGlu2/3; Kupferschmidt & Lovinger, 2015). Indeed, application of the mGlu2/3 agonist LY379268 (LY) induced a long‐lasting synaptic depression (Fig 4A and C; *black*) and also depressed synapses previously potentiated by IBMX (Fig 4A and C; *purple*) to levels similar to the depression by LY alone (Fig 4B). Striatal synapses can also be depressed through other presynaptic mechanisms, for example, activation of metabotropic gamma‐aminobutyric acid type B receptors (GABA_B_), which interfere with vesicular exocytosis in a SNAP‐25‐dependent manner (Manz *et al*, 2019) and reduce presynaptic calcium entry (Kupferschmidt & Lovinger, 2015). Application of the GABA_B_ agonist baclofen (BAC) depressed evoked EPSCs (Fig 4D–F; *black*); however, if synapses were potentiated by IBMX, subsequent BAC induced only a transient de‐potentiation (Fig 4D–F; *orange*). Thus, LY and BAC work through different presynaptic mechanisms which are differently affected by PDEs. This difference was even more pronounced when we reversed the order of application: While synapses were equally depressed by LY (*green*) and BAC (*blue*), the LY‐induced depression was merely reversed by subsequent inhibition of PDEs, whereas BAC‐induced depression turned into potentiation (Fig 4G–I). Taken together, our results indicate that PDEs limit VDCC engagement in the presynaptic terminals and their inhibition boosts VDCC‐dependent glutamate release.

Glutamatergic synaptic transmission in the striatum is also subject to neuromodulation, for example, through cholinergic interneurons and activation of muscarinic and/or nicotinic receptors on presynaptic terminals (Tanimura *et al*, 2019). However, we found no evidence that cholinergic modulation is involved in the IBMX‐induced potentiation of EPSCs (Appendix Fig S5A–C).

### Potentiation of synaptic release is mediated by cGMP, not cAMP signaling

**Figure 5:**
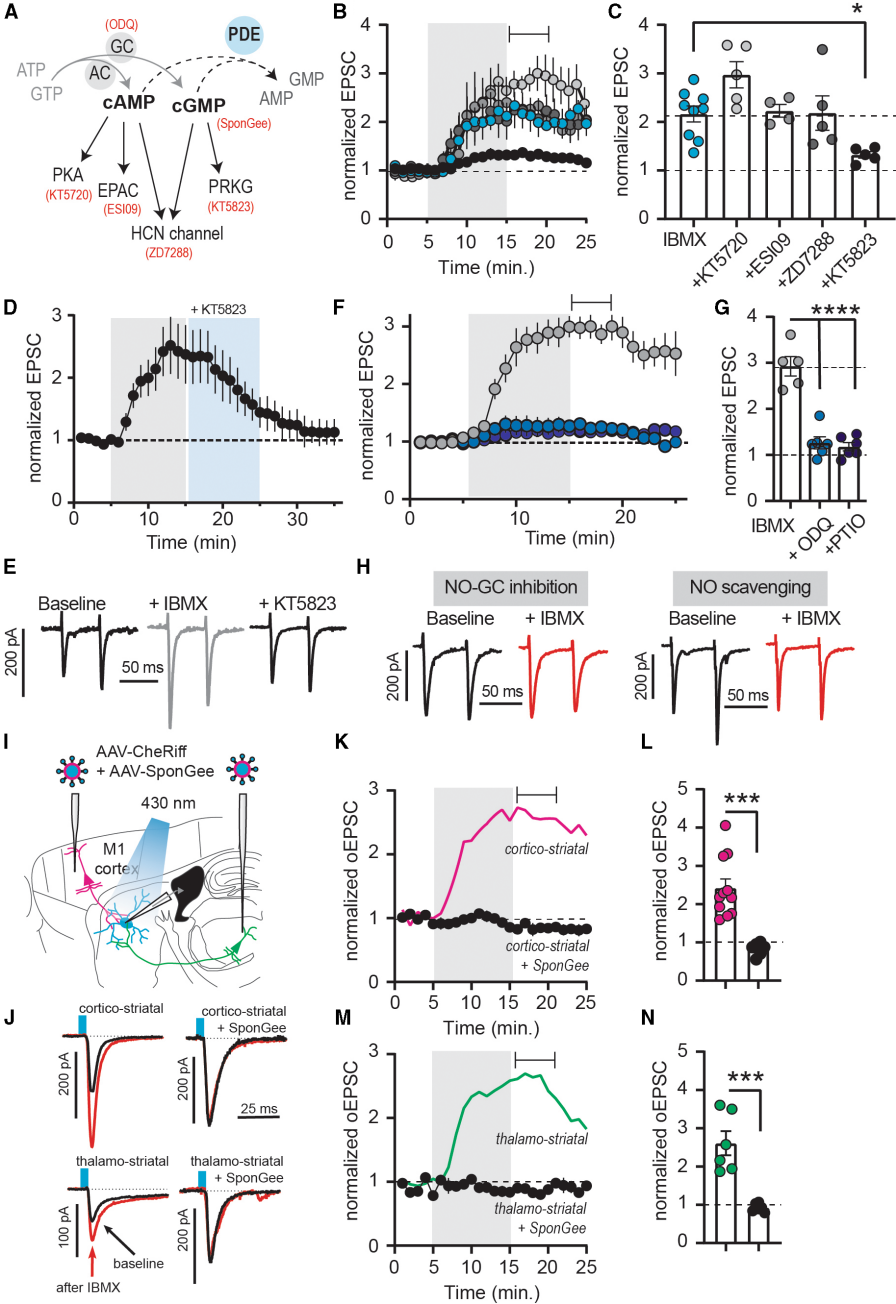
Cyclic GMP, not cAMP potentiates transmitter release in striatum AInhibition of PDE1 decreases both cAMP and cGMP hydrolysis and potentially activate downstream signaling *via* PKA, EPAC, PRKG, or HCN channels. In red are respective inhibitors or scavengers.BEPSCs with downstream inhibitors (see A and C) before and after IBMX (75 μM; gray shading). Mean ± SEM. Drugs were used at following concentrations: KT5720 0.5 μM, ESI09 15 μM, ZD7288 30 μM, KT5823 1 μM.CAveraged responses from the time window indicated in (B). *P < 0.05, ANOVA followed by Dunnett's multiple comparison test versus IBMX, *N* = 4–7. Mean ± SEM. IBMX data in (B) and (C) are replotted from Fig 1.DEPSCs with bath application of IBMX (75 μM), followed by PRKG inhibitor KT5823 (1 μM). Mean ± SEM. *N* = 7.EExample traces from (D).FEPSCs before and after IBMX (75 μM, gray shading), with either short (10 min, “IBMX”, *gray*) or long incubation (> 60 min) in NO‐GC inhibitor ODQ (10 μM, *light blue*), or long incubation in NO‐scavenger carboxy‐PTIO (> 60 min, 50 μM, *dark blue*). Mean ± SEM.GAveraged responses for the time window indicated in (F). ****P < 0.05, ANOVA followed by Dunnett's multiple comparison test versus IBMX, *N* = 5–6. Mean ± SEM.HExample traces from (F).INeurons in M1 or the PF co‐expressed CheRiff and the cGMP scavenger SponGee.JExample recordings before (*black*) and after (*red*) IBMX, controls from Fig 1.KEffect of IBMX on cortico‐striatal oEPSCs in the presence of SponGee (*black*). Control oEPSCs are replotted from Fig 1 (*magenta*). Mean ± SEM.LCorticostriatal oEPSCs averaged from the time indicated in (F). ***P < 0.001, unpaired *t*‐test, *N* = 11 and 6. Mean ± SEM.M, NAs (J, K) but thalamostriatal oEPSCs. Controls (*green*) are replotted from Fig 1. ***P < 0.001, unpaired *t*‐test, *N* = 6 and 6. Mean ± SEM.

Whereas some PDEs preferentially metabolize only one substrate, others, like PDE1, degrade both cAMP and cGMP. In principle, either cyclic nucleotide could be responsible for increased synaptic transmission under conditions of PDE1 block. Our prime candidate was cAMP, as it is an established regulator of synaptic transmission at other synapses and is known to modulate transmitter release through effects on VDCCs. There are three principal downstream targets of cAMP: protein kinase A (PKA), EPAC (exchange factor directly activated by cAMP), and HCN channels (Fig 5A). While any of these could be responsible for enhancing glutamate release after PDE inhibition, PKA seemed the most likely candidate due to similar involvement in other synapses (Chavez‐Noriega & Stevens, 1994; Huang & Hsu, 2006). To our surprise, blocking PKA with KT5720 did not prevent synaptic potentiation by IBMX (Fig 5B and C) and we confirmed this finding using two other inhibitors, H89 and cAMPS‐RP (Appendix Fig S6A–C). Of note, KT5720 showed a trend toward enhancement of the IBMX effect in some neurons. However, this effect was not consistent and not observed with the other PKA inhibitors. Similar to PKA, neither inhibition of EPAC nor HCN channels affected IBMX‐induced synaptic potentiation (Fig 5B and C). Inhibition of the cGMP‐dependent protein kinase 1 (PRKG) markedly attenuated the synaptic potentiation induced by IBMX (Fig 5B and C), indicating that cGMP‐ and not cAMP signaling is central to this effect. Interestingly, while inhibition of PRKG with KT5823 also reversed the IBMX‐induced potentiation of EPSCs (Fig 5D and E), it did not affect baseline synaptic strength (Appendix Fig S6D and E). This suggests that PRKG is not active at rest, but becomes activated when PDEs are inhibited, extending the duration of synaptic potentiation by altering VGCC conductance or the balance of the ready releasable pool (Hardingham *et al*, 2013).

One of the main sources of cGMP in the brain is nitric oxide (NO)‐sensitive guanylyl cyclases (NO‐GCs). To test whether the cGMP responsible for PRKG activation is produced by NO‐GCs, we performed recordings in the presence of NO‐GC inhibitor ODQ. Short application of ODQ (10 min) did not prevent the effect of IBMX on evoked EPSCs (Fig 5F, “IBMX,” *gray*). If slices were pre‐incubated in ODQ (60 min), however, the effect of PDE inhibition was markedly reduced (Fig 5F–H). Similar results were achieved after pre‐incubation (60 min) with NO scavenger carboxy‐PTIO (Fig 5F–H).

To validate the importance of cGMP, we made use of SponGee, a recently developed molecular scavenger protein that clamps cGMP to baseline levels (Ros *et al*, 2019). We expressed SponGee together with the channelrhodopsin CheRiff in M1 or PF (Fig 5I) and optogenetically evoked EPSCs in the dorsolateral striatum (Fig 5J). PDE inhibition was without effect on cortico‐ and thalamostriatal synapses when cGMP signaling was buffered by SponGee (Fig 5J–N), confirming our pharmacological results and demonstrating that presynaptic cGMP, not cAMP, controls the gain of striatal synapses.

### Presynaptic cGMP buffering alters release parameters

**Figure 6:**
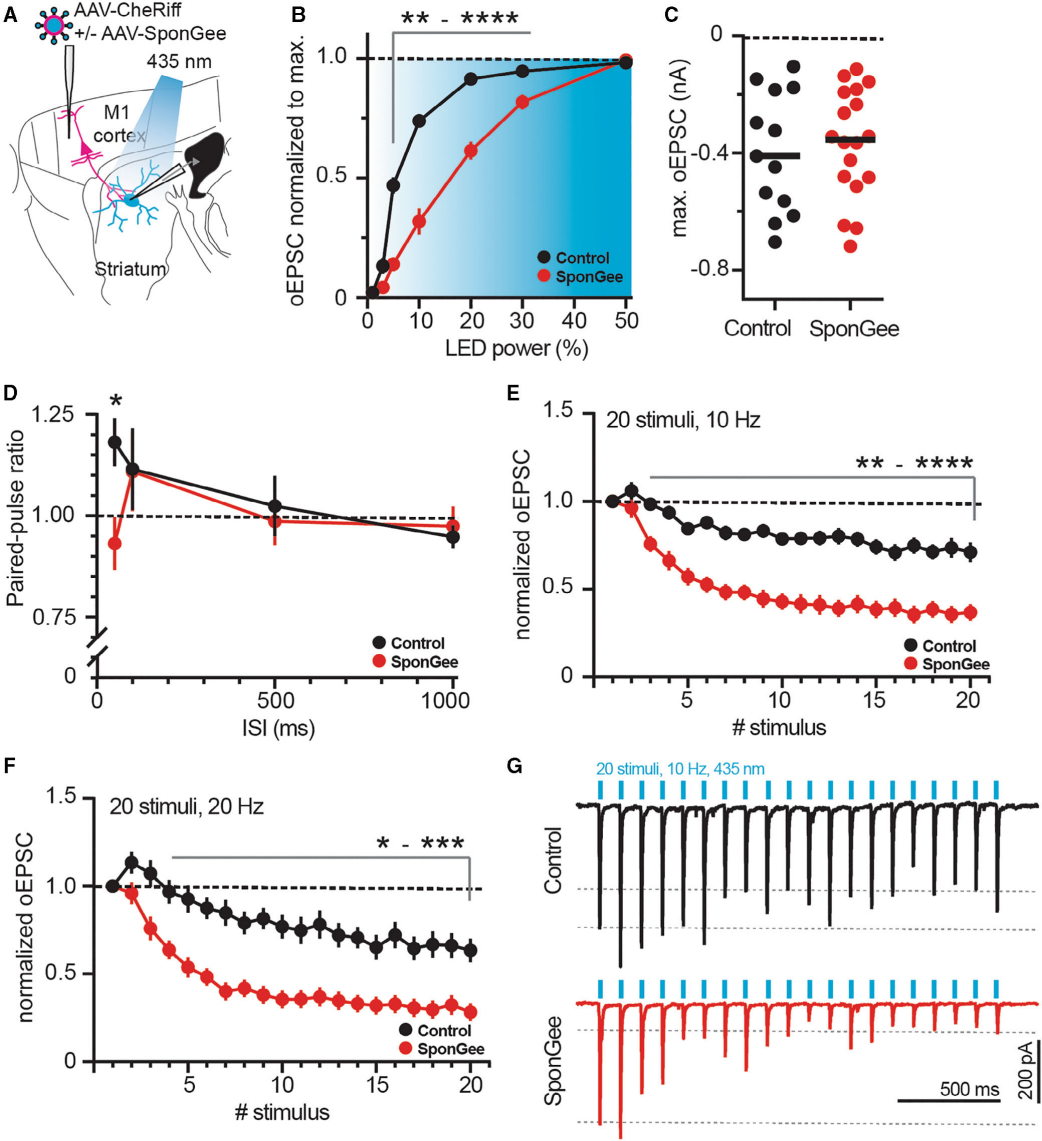
Scavenging cGMP with SponGee alters corticostriatal neurotransmitter release ASketch of experimental design. SPNs were patched in the striatum and oEPSCs evoked from cortical neurons with or without SponGee.BRight shift of the input–output relationship in the presence of SponGee. **P < 0.01, ***P < 0.001, ****P < 0.0001 two‐way ANOVA followed by Sidak's *post hoc* test, *N* = 13 and 18. Mean ± SEM.CMaximum evoked current is not changed by the presence of SponGee. *P* = 0.69, unpaired *t*‐test, *N* = 13 and 18.DPaired‐pulse ratios measured for different interstimulus intervals (ISI). *P < 0.05, two‐way ANOVA followed by Sidak's *post hoc* test, *N* = 13 and 18. Mean ± SEM.EAdaptation of oEPSCs for 20 consecutive stimuli given at 10 Hz. The oEPSC amplitudes are normalized to the respective first stimulus. **P < 0.01, ***P < 0.001, ****P < 0.0001, two‐way ANOVA followed by Sidak's *post hoc* test, *N* = 13 and 18. Mean ± SEM.FAs in (E) but with 20 stimuli given at 20 Hz. *P < 0.05 **P < 0.01, ***P < 0.001 two‐way ANOVA followed by Sidak's *post hoc* test, *N* = 13 and 18. Mean ± SEM.GExample traces for 10 Hz stimulation in (E).

Having observed that SponGee renders axonal terminals insensitive to IBMX, we wondered if scavenging cGMP would also alter baseline transmission at corticostriatal synapses. We patched SPNs after transducing M1 cortical cells with CheRiff or Cheriff and SponGee, and measured oEPSCs in response to brief light stimulation (Fig 6A). Even though the input–output curves are right‐shifted (Fig 6B), the maximally evoked oEPSC was not altered by the presence of SponGee (Fig 6C). We then looked at other presynaptic parameters by stimulating release with two pulses at different interstimulus intervals (ISIs). PPRs were largely the same; however, we observed a marked switch from facilitation to depression for the shortest measured interval of 50 ms (Fig 6D). To elaborate on this, we stimulated with a higher number of pulses at a higher frequency of 10 or 20 Hz. Under control conditions, trains of 20 pulses at either frequency showed an overall modest depression. Expression of SponGee and clamping cGMP, however, drastically altered these responses, resulting in a more pronounced depression of successive stimuli (Fig 6E–G).

### Presynaptic cGMP is critical for motor skill learning

**Figure 7:**
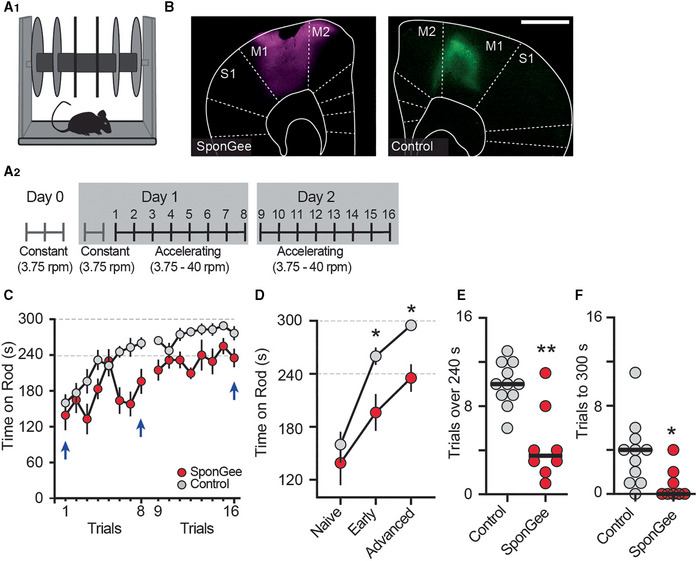
Preventing cGMP‐dependent presynaptic corticostriatal plasticity hampers motor learning in mice A1, A2Mice were trained on the rotarod. A2 shows time course of the motor skill learning paradigm, starting with a habituation on a constant speed on day 0, and an additional round of habituation on day 1. Mice were trained for eight trials on two consecutive days.BExample pictures from each group, either expressing AAV2/9‐synapsin‐SponGee (left) or a control AAV (AAV2/9 synapsin‐CheRiff‐Cerulean, right) in the M1 motor cortex. Scale bar 1 mm.CMean time spent on accelerating rod during each of eight trials on days 1 and 2. Control mice (gray) show progressive learning, that is, longer times on the accelerating rod, over both test days. Mice with SponGee (*red*) perform less well. Blue arrows indicate the time points compared in (D). Mean ± SEM.D“Naïve” is the first trial on the accelerating rotarod, “early” the performance on the last trial on day 1 and “advanced” on the last trial at the end of the second day of motor learning. *P < 0.05, two‐way ANOVA followed by Sidak's *post hoc* test, *N* = 11 and 8. Mean ± SEM.E, F(E) Number of trials that each mouse stayed on the rod for at least 240 s or (F) Until the cut‐off time of 300 s. *P < 0.05, **P < 0.01, unpaired *t*‐test, *N* = 11 and 8.

The corticostriatal pathway is important for motor skill learning, and it has been shown that axon terminals from the M1 motor cortex show heightened activity in the dorsolateral striatum when animals train on the accelerating rotarod (Kupferschmidt *et al*, 2017). We thus asked whether disrupting cGMP signaling in corticostriatal terminals would interfere with this type of learning, using bilateral expression of SponGee in M1 neurons (Fig 7A and B). We started with two control groups; mice that were not injected and mice that were transduced with a control AAV (AAV2/9‐synapsin‐CheRiff‐Cerulean) in M1. As the control groups showed equal performance on the accelerating rotarod, we pooled their data. All mice were familiarized with the nonaccelerating rotarod before the start of training. Importantly, at the start of training, there was no difference in the ability of control and SponGee‐expressing mice to remain on the rod, indicating the absence of strong motor or balance deficits (Fig 7C and D). On successive testing trials, control mice steadily improved in their ability to stay on the accelerating rod and attained almost perfect performance, that is, remaining for the full 5 min on the rod. In contrast, mice expressing SponGee in M1 neurons showed significantly worse performance, a deficit which remained on the second day of trials. This difference was also apparent when we evaluated the total number of trials each mouse remained on the rod for at least 240 s, or the number of trials that ended with the mouse still on the rod (Fig 7E and F). Less than 40% of the SponGee mice maintained their balance for the entire trial duration, whereas over 90% of the control mice achieved this skill level in at least one trial.

We additionally tested all SponGee mice and a subset of control mice in the open field and observed that spontaneous locomotion was not impaired in these mice (Appendix Fig S7A and B). These observations, together with the lack of difference during habituation and at the start of rotarod training, suggest that there is an impairment in motor skill learning, but no general motor deficit in the mice‐expressing SponGee in M1 cortical neurons. In summary, our results strongly suggest that cGMP controls presynaptic strength of striatal afferents through PRKG and VDCCs (Appendix Fig S8) and that regulation of corticostriatal synapses through cGMP is important for motor skill learning.

## Discussion

In many brain areas, such as in the hippocampus (Chavez‐Noriega & Stevens, 1994), cerebellum (Chen & Regehr, 1997), at the Calyx of Held (Kaneko & Takahashi, 2004), and in the prefrontal cortex (Huang & Hsu, 2006), cAMP potentiates synaptic transmission through increased presynaptic release. Thus, we were surprised that glutamate release at both corticostriatal and thalamostriatal synapses is controlled by cGMP signaling. This critical role of cGMP was unmasked by inhibiting PDE activity, suggesting that cGMP‐producing GCs are constantly active in presynaptic terminals producing cGMP, which is constantly metabolized by PDE1 (see Appendix Fig S8). Constitutive cGMP production and degradation appear energetically wasteful, but may indicate that in these terminals, rapid control of cGMP signaling is required.

Using PDE inhibitors, our experiments revealed the importance of cGMP in presynaptic regulation. However, complete block does not mirror a situation likely occurring in a physiological context. What could be possible naturally occurring regulators of this system? Our current working hypothesis puts PDE1 into a central position, because its enzymatic activity can be bidirectionally modulated (Appendix Fig S8): Ca^2+^ and Calmodulin (CaM) increase its activity, while phosphorylation by PKA and CaM‐kinase II (CaMKII) reduces its binding affinity (Hashimoto *et al*, 1989; Florio *et al*, 1994; Omori & Kotera, 2007). We postulate that PDE1 activity is high during phases of strong presynaptic activity, keeping cGMP low. During phases of low presynaptic activity, Ca^2+^/CaM levels are low, resulting in reduced PDE1 activity, elevation of cGMP levels, and increased transmitter release. Coincident activation of PKA/CaMKII by neuromodulatory activity would further enhance this effect (Appendix Fig S8). PDE1, through regulating cGMP, seems to regulate glutamate release in a homeostatic fashion, reducing the gain at the most active inputs. Similarly, the production of NO by neuronal NO synthase is under physiological conditions Ca^2+^‐dependent and triggered by CaMKII‐mediated phosphorylation (Lee & Stull, 1998; Zhou & Zhu, 2009).

Controlling excitatory input to the basal ganglia at the level of individual synaptic terminals has many advantages. It allows sculpting the pattern of glutamatergic excitation based on the level of ongoing presynaptic input neuron activity, and in addition, provides numerous entry points for powerful neuromodulatory control. This represents an additional level of information processing that takes place before the integration of excitatory inputs in SPN dendrites.

Is cGMP‐mediated presynaptic potentiation unique to striatal synapses? One important activator of GCs is nitric oxide (NO). In the striatum, NO is produced in large quantities, mostly by interneurons, and reportedly induces both postsynaptic depression (Calabresi *et al*, 1999; Rafalovich *et al*, 2015) and increased responsiveness of SPNs to cortical inputs (West & Grace, 2004; Tseng *et al*, 2011). Nitric oxide has also been reported to regulate presynaptic functions in other brain areas (Hardingham *et al*, 2013), with most data being acquired in the hippocampus and neocortex. Initial studies, however, reported that in the hippocampus, injection of cGMP into the presynapse is not sufficient to alter synaptic transmission, but enhances responsiveness to weak stimuli, and under some circumstances promotes long‐term potentiation (Zhuo *et al*, 1994a, 1994b; Arancio *et al*, 1995). More recent studies in hippocampus and somatosensory cortex have largely confirmed that cGMP analogs do not affect synaptic transmission (Taqatqeh *et al*, 2009; Neitz *et al*, 2014; Wang *et al*, 2017; but see (Neitz *et al*, 2011). A small cGMP‐dependent potentiation of glutamatergic release occurs in the ventrolateral medulla (Huang *et al*, 2003), and transmitter release in the chick ciliary ganglion is potentiated through a PRKG‐dependent mechanism (Yawo, 1999). Thus, although effects of cGMP signaling on synaptic transmission are not unheard of, the strong dependence of striatal synaptic strength on cGMP and weak sensitivity to cAMP we observed is exceptional.

The striatum receives synaptic inputs from multiple brain regions, including a vast array of glutamatergic projections from cortical and thalamic areas. Among the cortical inputs, the majority originate in the deep layers of primary motor and somatosensory cortices, whereas the PF is the main thalamic nucleus projecting to the striatum (Wall *et al*, 2013). Our data suggest that NO‐GCs, PDE1, and PRKG are active in the terminals arising from these regions, and there is indeed evidence from expression studies supporting this. PDE1 is widely expressed throughout the thalamus (Polli & Kincaid, 1994; Hepp *et al*, 2007; Kelly *et al*, 2014). However, the brain‐wide highest expression is found in the cortex, with specific enrichment of PDE1A in the deep cortical layers (Kelly *et al*, 2014), from where projections to the striatum originate. PRKG2 is generally expressed throughout the rodent brain, but highest mRNA levels are found across the thalamus (El‐Husseini *et al*, 1995, 1998; Geiselhoringer *et al*, 2004; Demyanenko *et al*, 2005). It is furthermore present in the cortex, including the motor and sensory areas, where mRNA levels differ depending on the layer (Geiselhoringer *et al*, 2004; Werner *et al*, 2004; Demyanenko *et al*, 2005). While PRKG2 mRNA seems to be generally enriched in the superficial layers, it is also found in the critical deep cortical layers (de Vente *et al*, 2001). Lastly, NO‐GC is found in both thalamus and cortex of rodents (Gibb & Garthwaite, 2001; Pifarre *et al*, 2007), specifically in the PF (Furuyama *et al*, 1993) and again the deep cortical layers (Furuyama *et al*, 1993; Giuili *et al*, 1994). These studies lack, however, cell‐type specificity and sufficient resolution to directly show PDE1, PRKG, and NO‐GC in the cortico‐ and thalamostriatal terminals. Nevertheless, based on the high levels and density of detected mRNA in the regions and layers of origin, it seems likely that all three are expressed in the projection neurons of these areas. It remains to be seen whether glutamatergic projections originating from cortical areas outside M1, thalamic nuclei outside PF, or other brain regions, for example, the amygdala, are similarly controlled by cGMP.

We found not only evoked but also spontaneous transmitter release to be enhanced after inhibition of PDEs. Although one does not necessarily predict the other, there are several studies showing that altering presynaptic Ca^2+^ influences the mEPSCs frequency. For example, BDNF leads to an increase of mEPSCs in the hippocampus depending on both presynaptic Ca^2+^ influx and release from internal stores (Amaral & Pozzo‐Miller, 2012; Schneider *et al*, 2015), whereas chelating Ca^2+^ with BATPA‐AM reduces mEPSC frequency by altering presynaptic VDCC mobility (Schneider *et al*, 2015). Importantly, an increase of cGMP, PRKG activation, and number of presynaptic synaptophysin puncta underlie the glutamate‐induced increase of mEPSCs in cultured hippocampal neurons (Wang *et al*, 2005), in line with previous findings showing that direct application of a cGMP analog increases the frequency of mEPSCs through a presynaptic mechanism (Arancio *et al*, 1995).

Corticostriatal synapses are of crucial importance in various disorders, including those with characteristic motor and motor skill learning deficits, such as Parkinson's and Huntington's disease (Shepherd, 2013). Previous work has demonstrated that presynaptic activity of corticostriatal terminals in the dorsolateral striatum (originating from M1) potentiates in mice during motor skill learning (Kupferschmidt *et al*, 2017). Although we have not directly demonstrated that cGMP‐dependent synaptic plasticity occurs *in vivo*, our data show that interfering with cGMP in cortical neurons impedes motor learning. Our interpretation is that expression of SponGee in M1 neurons affected rotarod learning specifically by interfering with cGMP‐dependent regulation of synapses onto SPNs—either by preventing their cGMP‐dependent potentiation (Fig 5) or by reducing glutamate release (Fig 6) or both. In addition, it is possible that cGMP‐dependent mechanisms acting at other M1 synapses or intracellular processes contributed to the learning deficit. Whether cGMP‐dependent plasticity is also disturbed in disease models with motor skill learning deficits will be an interesting question for future studies.

In clinical trials, inhibitors of PDE1 improved cognitive function in healthy individuals, however not in patients with Alzheimer's disease, and studies of patients with Schizophrenia and Parkinson's disease are under way (Kelly *et al*, 2014; Heckman *et al*, 2018). The beneficial effects of PDE1 inhibition were thought to manifest through vasodilation or regulation of postsynaptic signaling downstream of the dopamine D1 receptor (Heckman *et al*, 2018). We show that PDE1 is also a critical regulator of presynaptic release in the striatum, therefore offering a new perspective for targeting PDE1 and cGMP signaling in the wide range of neurological diseases associated with corticostriatal dysfunction, ranging from movement to mood disorders.

## Materials and Methods

### Animals

For the experiments, we used C57BL6/N wild‐type and heterozygous BAC‐*adora2a*‐Cre transgenic mice as well as their nontransgenic littermates. The animals were bred in‐house at the University Medical Center Hamburg‐Eppendorf and kept group‐housed with their littermates on a 12 h light/dark cycle with access to food and water *ad libitum*. Both male and female mice were used and at least 8–10 weeks old at the start of the experiment. All experiments were conducted in accordance with the European Directive 2010/63/EU and were approved by the local authorities of the City of Hamburg (Behörde für Justiz und Verbraucherschutz; Lebensmittelsicherheit und Veterinärwesen).

### Acute brain slices

Mice were anesthetized with CO_2_ before rapid decapitation. The brains were dissected in ice‐cold choline solution, containing (in mM): choline‐chloride (110), KCl (2.5), NaH_2_PO_4_ (1.25), NaHCO_3_ (25), MgCl_2_ (7), CaCl_2_ (0.5), Glucose (25), sodium‐ascorbate (11.6), sodium‐pyruvate (3.1). The osmolarity of the choline solution was typically 310 mOsm/l, with a pH of 7.4. Oxygenation and pH were kept through constant bubbling with 95% O_2_ and 5% CO_2_. Parasagittal slices of 275 μm thickness were cut on a vibratome (Leica 1000 S). After cutting, slices were transferred into a holding chamber with regular artificial cerebrospinal fluid (aCSF), containing (in mM): NaCl (125), KCl (2.5), NaH_2_PO_4_ (1.25), NaHCO_3_ (26), MgCl_2_ (1), CaCl_2_ (2), Glucose (10). The aCSF was constantly bubbled with 95% O_2_ and 5% CO_2_ (pH 7.4, 305 mOsm/l). Acute brain slices were kept at 34°C for at least 45 min before the start of experiments.

### Electrophysiology and optogenetic stimulation

Brain slices were transferred into a recording chamber, superfused at a rate of 2.5–3 ml/min and experiments were performed at 30–31°C. Picrotoxin (50 μM) was added to the bath for all EPSC recordings to block GABA_A_ currents. Patch pipettes were pulled from borosilicate glass and had a resistance of 3–5 MΩ. Internal solution contained (in mM) K‐gluconate (135), HEPES (10), MgCl_2_ (4), Na_2_‐ATP (4), Na‐GTP (0.4), Na_2_‐phophocreatine (10), L‐ascorbic acid (3), EGTA (0.2). Internal solution had pH 7.2 and 295 mOsm/l. SPNs in the dorsolateral striatum were patched and whole‐cell recordings were performed using an Axopatch 200B or Mulitclamp 700B amplifier (Axon Instruments, Inc.), National Instruments A/D boards and Ephus software (Suter *et al*, 2010). SPNs were identified by soma size and typical firing patterns in response to somatic current injection (see Appendix Fig S2). In a subset of experiments, iSPNs and dSPNs were identified based on green or red somatic fluorescence and the typical electrophysiological profile. In line with previous reports (Gertler *et al*, 2008; Fieblinger *et al*, 2014), dSPNs were less excitable than iSPNs (Appendix Fig S2). EPSCs were electrically evoked using a monopolar electrode placed between the recorded cell and the cortex. Pulses were given using a constant current stimulator (IS4, Scientific Devices) at 0.05 Hz with 0.1 ms duration. For paired‐pulse analysis, two pulses were given with 50 ms interstimulus interval. The ratio was then calculated as the amplitude of the second pulse divided by the amplitude of the first pulse. At this interval, corticostriatal synapses typically show a PPR > 1. During the recordings, SPNs were held at −70 mV. Liquid junction potentials were not corrected for. For each recording, a stable baseline of 5–10 min was acquired and throughout the experiments; EPSC amplitudes were analyzed. For statistical comparison of drug effects, EPSC amplitudes were averaged over a time window of 5 min. For miniature EPSC recordings, TTX (1 μM) was added to the bath. SPNs were held at −70 mV, and for each cell, 90 s were analyzed before and after the application of IBMX.

Excitatory inputs expressing the channelrhodopsin CheRiff were stimulated optogenetically through the objective (Olympus, 60×, 0.9 NA), using brief pulses (2–5 ms) of 430 nm light (Prizmatix, Mic‐LED‐430) of ~1–12 mW/mm^2^ depending on the response strength. For both electric and optogenetic stimulation, the intensity was adjusted to induce baseline EPSCs of *circa* 50–300 pA. EPSC recordings were normalized to the 5–10 min baseline recording. To probe properties of SponGee‐expressing synapses (Fig 6), we delivered 435 nm light pulses (CoolLED, pE‐4,000, 2 ms pulses) through the objective (Olympus LUM Plan FL N 60×, 1.0 NA). Recordings were discarded if the access resistance at the end of the experiment was altered by > 20%. Data analysis was done in Matlab and Clampfit 10.7 (Molecular Devices).

### Slice pharmacology

The following drugs and chemicals were used: IBMX (75 μM, 10 min), LY379268 (200 nM, 5 min), Baclofen (3 μM, 10 min), KT5720 (500 nM, in bath), H89 (10 μM, in bath), cAMPS‐Rp (20 μM, in bath), ESI09 (15 μM in bath), ZD72885 (30 μM in bath), MMPX (10 μM, 10 min), rolipram (1 μM, 10 min), papaverine (10 μM, 10 min), BAY 667550 (2 μM, 10 min), KT5823 (1 μM, 10 min or in bath), CdCl_2_ (100 μM, in bath), ODQ (10 μM, in bath plus pre‐incubation), carboxy‐PTIO (50 μM, in bath plus pre‐incubation), scopolamine (10 μM, in bath), and mecamylamine (10 μM, in bath). IBMX, Baclofen, and ZD72885 were purchased from HelloBio, Papaverine from Sigma‐Aldrich, carboxy‐PTIO from SantaCruz, and all other compounds from Tocris.

### Stereotactic injections

Mice were put under analgesia (buprenorphine, 0.05 mg/kg bw) and anesthesia and placed into a stereotaxic frame (Stereo, InjectoMate, Neurostar). Anesthesia was maintained using 1.5% isoflurane in O_2_, and a heating pad was put under the animal to avoid hypothermia. Fur and skin were carefully removed and craniotomies were performed using an automated drill. Viral vector particles were injected through a fine pulled glass capillary, with an injection speed of 100 nl/min. After the injection, the capillary was left in place for an additional 5 min. The skin was stitched at the end of the surgery and animals were provided with postoperative analgesic treatment (Caprofen, 5 mg/kg bw). We waited 3–4 weeks for the viral expression before starting the experiments.

The following viral constructs were used: AAV2/9‐syn‐CheRiff‐cerulean (1 × 10^12^ vg/ml), AAV2/9‐syn‐iGluSnFR‐2A‐tdimer2 (1.5 × 10^13^ vg/ml), AAV2/9‐syn‐SponGee (8.6 × 10^13^ vg/ml), AAV2/9‐Ef1a‐DO_DIO‐tdTomato_EGFP (2.3 × 10^13^ vg/ml), AAV2/9‐syn‐jGCaMP7b (4.4 × 10^14^ vg/ml). Injections were performed at the following coordinates: M1 cortex: AP +1.7, ML −1.5, DV −1.9 and −1.25 (250 nl per site); PF thalamus: AP −2.3, ML −0.6, and DV −3.5 (500 nl); striatum: AP +1.0, ML −2.1, DV −3.5 and −3.0 (350 nl per site). The viruses were prepared at the UKE vector facility, based on the following plasmids: RRID:Addgene_51697 (CheRiff; Hochbaum *et al*, 2014); RRID:Addgene_106174 (iGluSnFR; Marvin *et al*, 2018); RRID:Addgene_134775 (SponGee; Ros *et al*, 2019); RRID:Addgene_37120 (DO_DIO‐tdTomato_EGFP; Saunders *et al*, 2012); RRID:Addgene_104489 (jGCaMP7b; Dana *et al*, 2019).

### Two‐photon imaging

For fast 2‐photon imaging, we used a customized version of the Rapid3Dscope (Rapp OptoElectronic GmbH), a large field of view two‐photon microscope equipped with resonant‐galvo‐galvo scanners. The microscope is controlled by the open‐access software ScanImage 2017b (Pologruto *et al*, 2003). To image jGCaMP7b or simultaneously excite both the membrane‐bound iGluSnFR and the cytoplasmic tdimer2, we employed a pulsed Ti:Sapphire laser (Chameleon Ultra II, Coherent) tuned to 930 nm. Red and green fluorescence was detected through upper and lower detection paths with photomultiplier tubes (PMTs, H7422P‐40SEL, Hamamatsu). The lower detection has an oil immersion condenser (1.4 NA, Olympus), a 560 DXCR dichroic mirror, and 525/50 (green) and 607/70 (red) emission filters (Chroma Technology). The upper detection has the objective (Nikon CFI75 LWD, 16×, 0.8 NA; or Leica HC APO L, 20×, 1.0 NA), the main short‐pass dichroic (Chroma ZT775sp‐2p), and a dichroic mirror (Chroma T565lpxr) with red and green emission filters (Chroma ET525/70m‐2p, Chroma ET605/70m‐2p). Excitation light was blocked by short‐pass filters (Chroma ET700SP‐2P). For extracellular synaptic stimulation, a glass monopolar electrode filled with extracellular solution was placed in the striatum. Single 0.2–0.5 ms pulses were delivered using an ISO‐Flex stimulator (A.M.P.I.) at 0.033 Hz. Pulse strength was carefully increased until obtaining a stable fluorescence response to the stimulus. For glutamate imaging, overview images (437 × 437 μm) were acquired with resonant scanning at 30 Hz to detect release sites. We then reduced our imaging field of view to a 128 × 128‐pixel (73 × 73 μm^2^) area close to the electrode tip where we recorded fluorescence at 110 Hz, averaging two frames (actual rate: 55 Hz). For calcium imaging, the field of view around the electrode measured 256 × 256 pixel (90 × 90 μm^2^) averaging four frames at a final rate of 15 Hz. Triggering of laser scanning and acquisition along with electrical stimulation was done by the electrophysiology software Wavesurfer yoked to ScanImage. The resonant scanner was engaged before acquisition start, and laser power was controlled by a Pockel cell (Conoptics). Electronic shutters (Uniblitz) were in place at the beam path and before PMTs to protect the user, sample, and PMTs between acquisition periods. Images were analyzed using ImageJ (NIH).

### Behavior

All mice were habituated to the rotating rod (3.2 cm diameter, 3.75 rotations per minute (rpm), TSE systems) for two consecutive 2‐min trials with an inter‐trial interval (ITI) of 10 min the day before beginning the accelerating rotarod. Any falling mice were placed back on the rod until the 2 min were over. The next day the mice were again put on the slow constantly rotating rod for 2 min. No mice fell off the rod during this habituation test trial. The mice were then tested in eight consecutive trials with accelerating rotation (from 3.75 to 40 rpm over 300 s), with an ITI of 10 min. A trial stopped when a mouse fell off the rod, if it held onto the rod for two consecutive rotations without running, or after 300 s. On the second day, mice again performed in eight consecutive trials, 10 min ITI, on the accelerating rotarod. The tests were performed under red light by an experimenter blind to the treatment and following the ARRIVE guidelines. One control mouse was excluded as it showed no improvement at all in the task. Injection and expression of the virus was verified in all animals. The AAV2/9‐syn‐CheRiff‐Cerulean virus was used for some control mice. To test general locomotion deficits, mice were put in an open field (50 × 50 cm) arena and activity was recorded for 60 min under dim light conditions (15–20 lux). Movement speed and distance traveled were analyzed in 5 min bins using EthoVision (Noldus).

### Quantification and statistical analysis

All data are displayed as mean ± standard error of the mean. Statistical analysis was performed using GraphPad Prism (Version 8.3.0) as specified in Appendix Table S1. Significance level was set at *P* < 0.05.

## Author contributions

**Tim Fieblinger:** Conceptualization; data curation; formal analysis; supervision; funding acquisition; investigation; visualization. **Alberto Perez‐Alvarez:** Data curation; investigation. **Paul J Lamothe‐Molina:** Data curation; investigation. **Christine E Gee:** Supervision; funding acquisition. **Thomas G Oertner:** Supervision; funding acquisition.

In addition to the CRediT author contributions listed above, the contributions in detail are:

Conceptualization: TF; patch clamp experiments: TF; 2‐Photon Imaging: AP‐A, TF; stereotactic surgeries: TF, PJL‐M; behavior tests: PJL‐M, TF; Analysis and Visualization: TF; Supervision: TF, CEG, TGO; funding: TF, CEG, TGO; Writing: TF wrote the initial draft with inputs from all the authors.

## Disclosure and competing interests statement

The authors declare that they have no conflict of interest.

## Supporting information




Appendix

## Data Availability

Data are available on reasonable request from the corresponding authors.
