# Supplementary material for: Presynaptic cGMP sets synaptic strength in the striatum and is important for motor learning
Source: EMBO Rep. 2022 Jun 23;23(8):e54361. doi: 10.15252/embr.202154361 (PMC9346481; doi:10.15252/embr.202154361)
Supplement: Supplementary file 1 — Appendix [file EMBR-23-e54361-s001.docx]

Presynaptic cGMP sets synaptic strength in the striatum and is important for motor learning

Tim Fieblinger^1*^, Alberto Perez-Alvarez^1^, Paul J. Lamothe-Molina^1^, Christine E. Gee^1^, Thomas G. Oertner^1^

^1^ Institute for Synaptic Physiology, University Medical Center Hamburg-Eppendorf, 20251 Hamburg, Germany

^*^ corresponding author: tim.fieblinger@zmnh.uni-hamburg.de

Appendix Figures and Table

Appendix Figures S1 ………………………………………………………………………………………………… page 2

Appendix Figures S2 ………………………………………………………………………………………………… page 3

Appendix Figures S3 ………………………………………………………………………………………………… page 4

Appendix Figures S4 ………………………………………………………………………………………………… page 5

Appendix Figures S5 ………………………………………………………………………………………………… page 6

Appendix Figures S6 ………………………………………………………………………………………………… page 7

Appendix Figures S7 ………………………………………………………………………………………………… page 8

Appendix Figures S8 ………………………………………………………………………………………………… page 9

Appendix Table S1 ………………………………………………………………………………………………… page 10

Appendix Figures


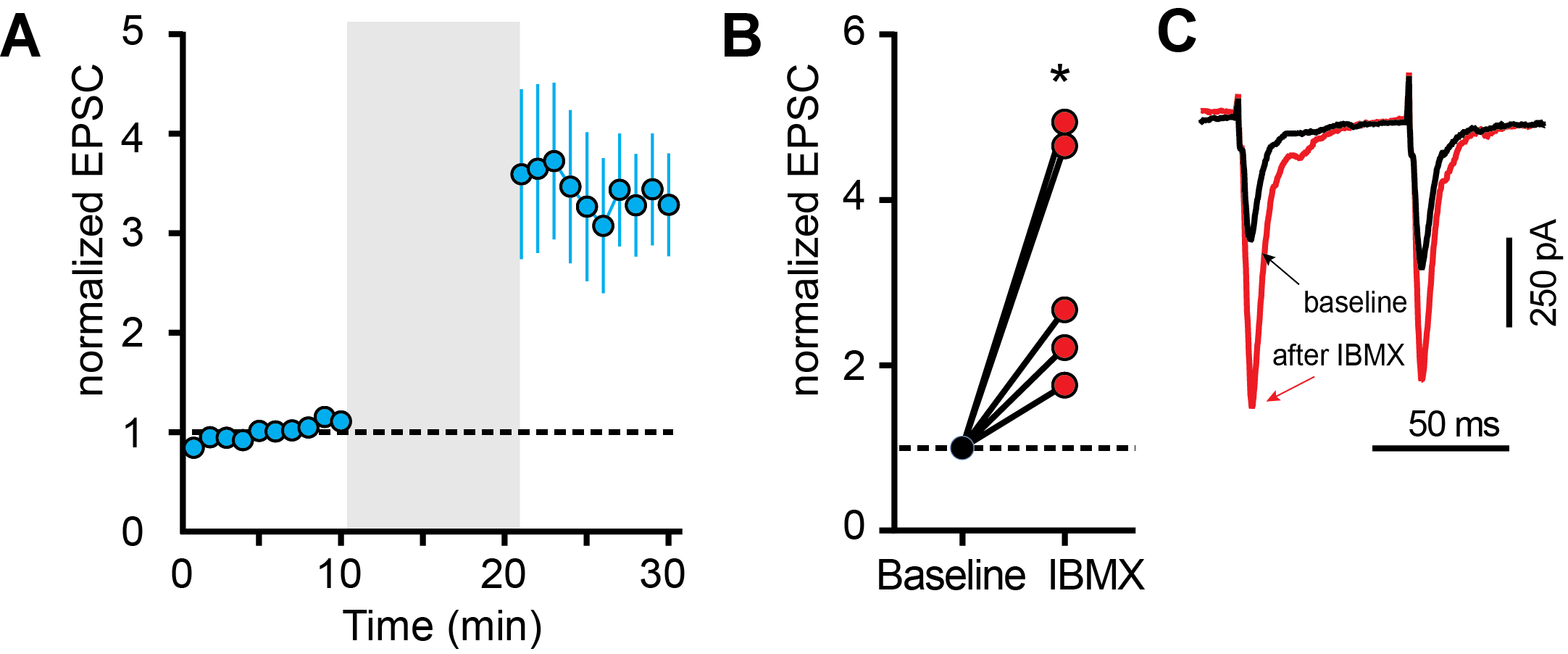


Appendix Fig S1. Synaptic potentiation by inhibition of phosphodiesterases does not require active synapses.

A After acquisition of a stable baseline, synaptic inputs were not stimulated during the application of IBMX (75 µM, gray area). Stimulation was re-started with the beginning of the IBMX washout. Mean ± SEM.

B EPSC amplitudes are compared to their respective baseline after IBMX. Data points represent averaged responses per cell during the ten-minute baseline and first five minutes of IBMX washout. * p < 0.05, paired t-test, N = 5.

C Example trace of a neuron recorded in (A).


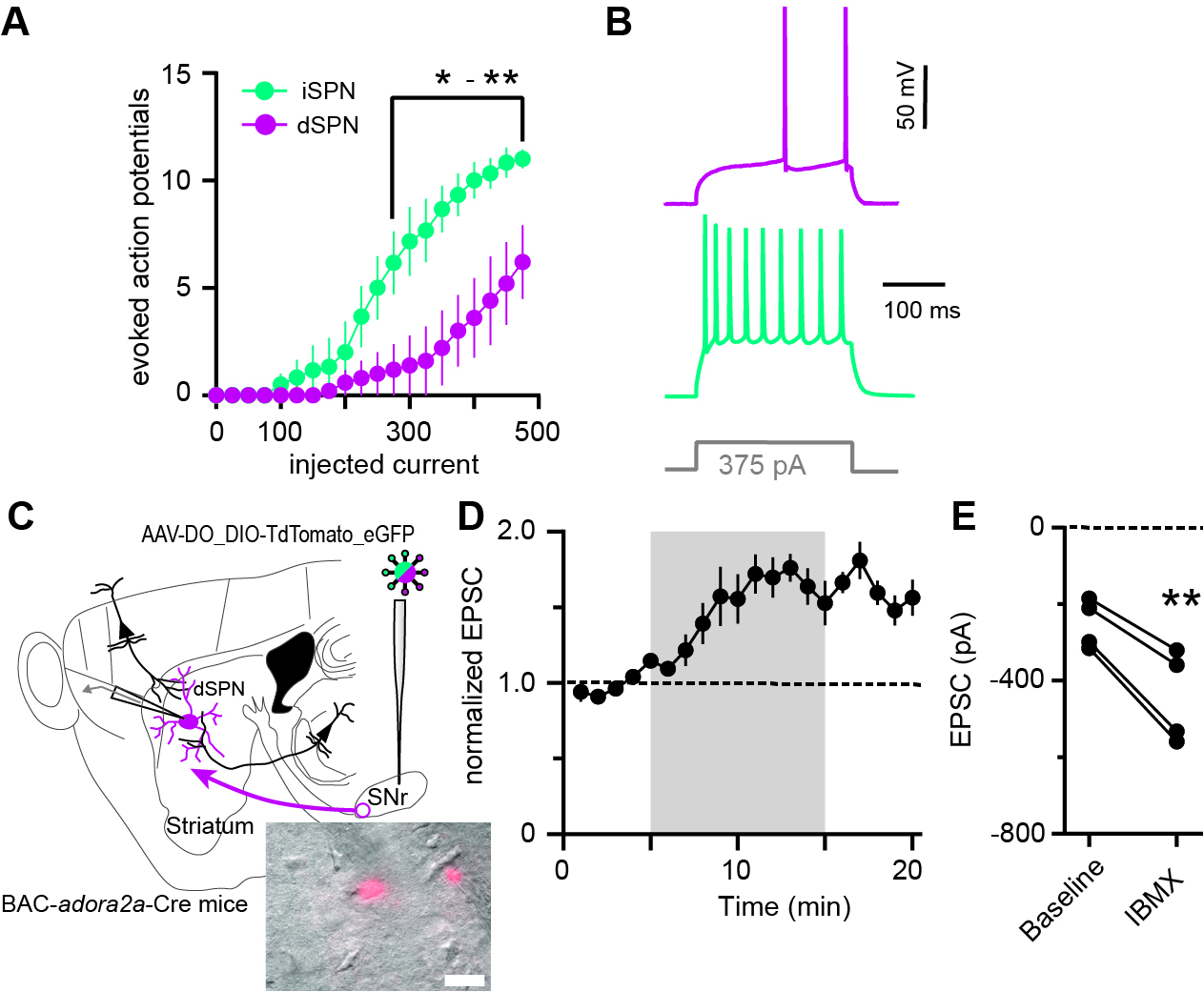


Appendix Fig S2. Segregation of iSPN and dSPN and recordings of retrogradely labeled dSPNs.

A Intrinsic excitability was measured by increasing current step injections and the resulting number of action potentials is plotted for dSPNs and iSPNs as defined by fluorescence (Fig. 1). * p < 0.05, ** p < 0.01, Two-Way RM ANOVA followed by Sidak’s multiple comparison test, N =5 and 6. Mean ± SEM.

B Representative responses for a 375 pA current injection are shown. In the iSPN (*green*), this stimulation evokes much more action potentials than in the dSPN (*magenta*).

C A retrogradely transported AAV-DO_DIO-tdTomato_eGFP was injected into the substantia nigra pars reticulata (SNr) of BAC-*adora2a*-CRE mice. Inset shows tdTomato-positive dSPNs in the dorsal striatum. No eGFP positive cells were observed (2 mice). Scale bar: 20 µm

D Time course of EPSC amplitude. Application of IBMX (75 µM) is indicated by the gray area. N = 4. Mean ± SEM.

E EPSC amplitude at baseline and after IBMX application. ** p < 0.01 paired t-test, n = 4 neurons (2 mice).


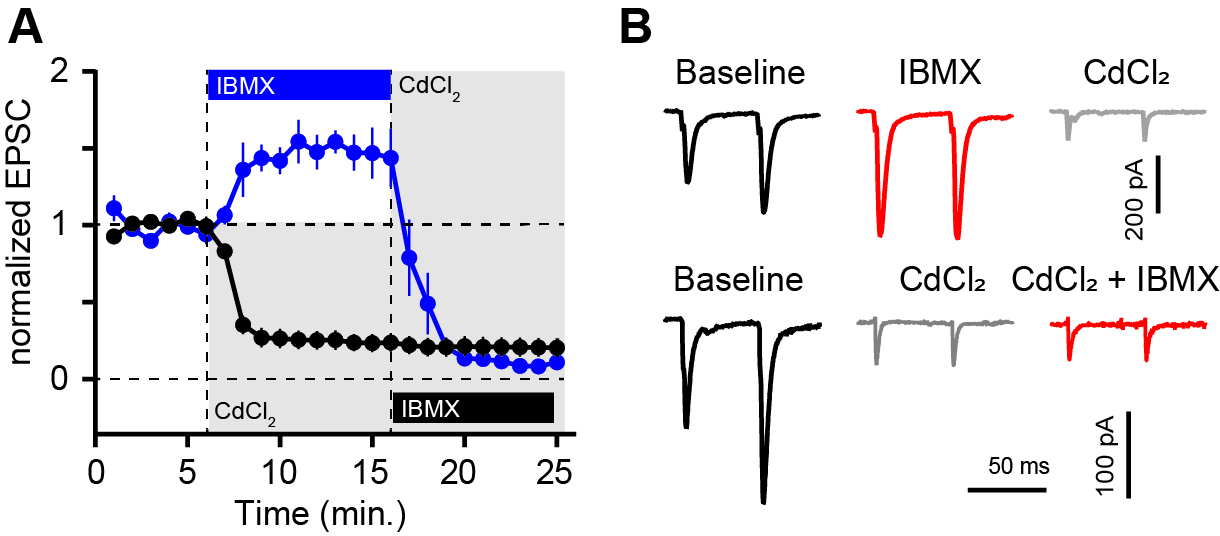


**Appendix** **Fig S3. Block of VDCCs prevents and reverts potentiation by inhibition of phosphodiesterases.**

A Synaptic potentiation was either first potentiated (IBMX, 75 µM; time indicated by bar; *blue*) and then continued to record in the presence of CdCl_2_ (100 µM, gray shade), or vice versa, EPSCs were first recorded in CdCl_2_ (gray shade) before IBMX was added (75 µM; indicated by bar; *black*). N = 5 and 7. Mean ± SEM.

B Example traces for both conditions, as in *(A)*.

**
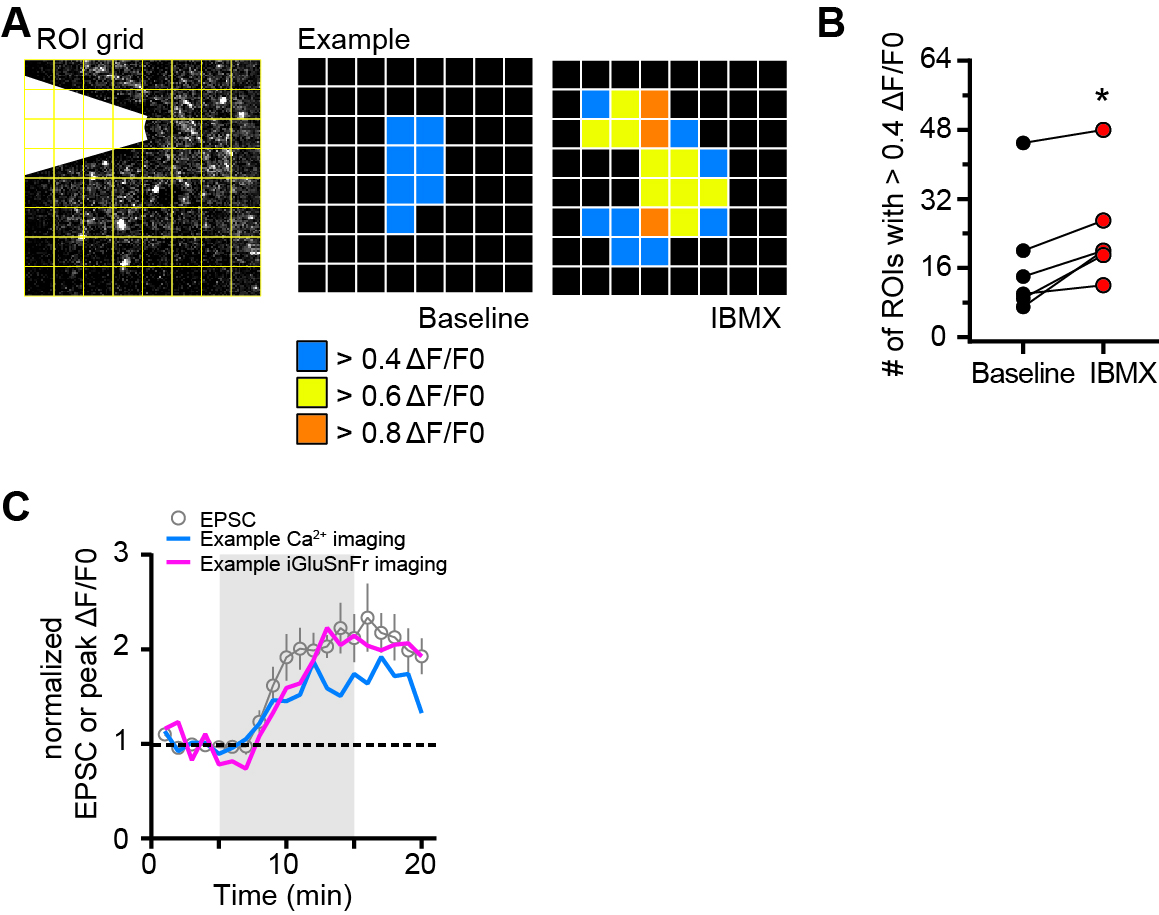
**

**Appendix Fig S4. Phosphodiesterase inhibition unlocks more glutamate release sites and time course comparison.**

A Example of region of interest (ROI) analysis. An 8 x 8 ROI grid was used to divide the field of view and ROIs with a peak > 0.04 ΔF/F0 were counted before and after IBMX (75 µM).

B Quantification of the number of ROIs with peaks. * p < 0.05, paired t-test, N = 6

C Time course example for presynaptic Ca^2+^- and glutamate imaging. Application of IBMX (75 µM) is indicated by gray area. Plotted are evoked EPSCs (gray, *from Fig. 2B,* Mean ± SEM*,* N = 8) and an representative example time course of the peak ΔF/F0 from the jGCaMP7b (*blue*) and iGluSnFR (*magenta*) experiments.


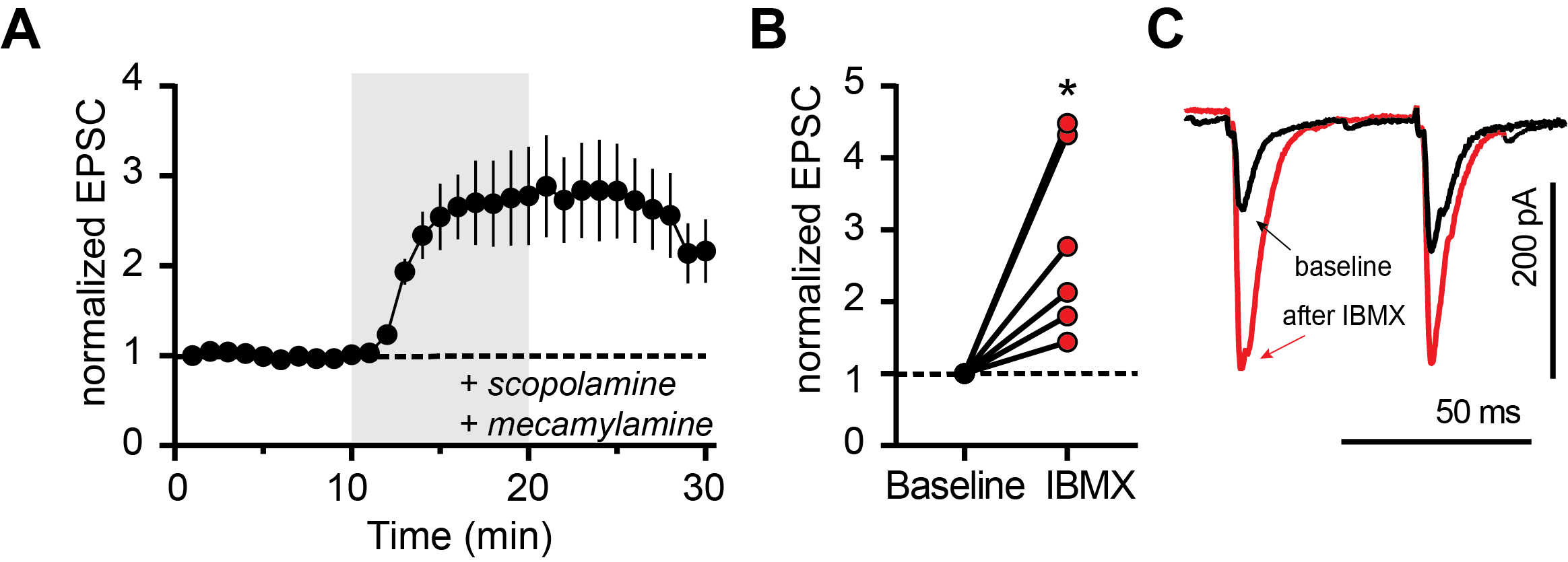


Appendix Fig S5. Synaptic potentiation is not mediated by cholinergic interneurons.

A Time course of EPSC recordings in the presence of scopolamine (10 µM) and mecamylamine (10 µM), to block nicotinic and muscarinic signaling, and the response to PDE-inhibition. Gray box indicates application of IBMX. Mean ± SEM.

B Quantification of the effect of IBMX on evoked EPSCs. Data points represent averaged responses per cell during the ten-minute baseline and a five-minute window after the IBMX application. * p < 0.05, paired t-test, N = 6.

C Example recording from a neuron in (A).


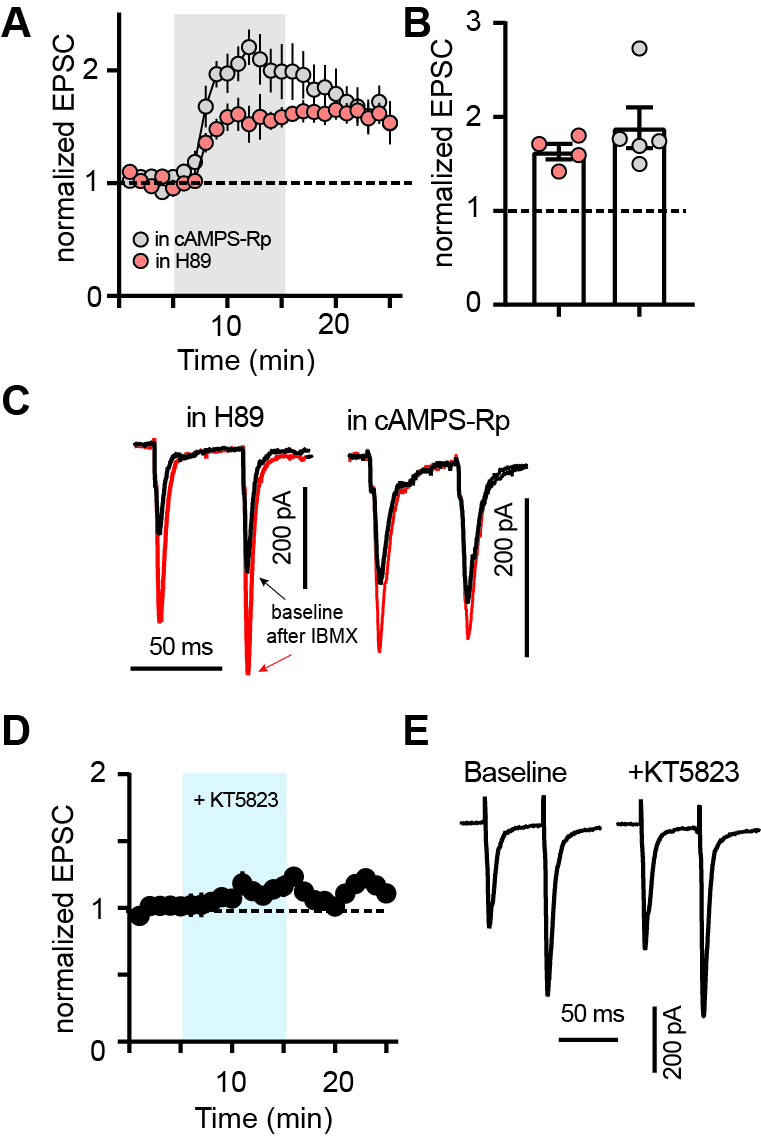


Appendix Fig S6. Synaptic potentiation is not prevented by two different PKA inhibitors.

A Time course of EPSC recordings in the presence of either cAMPS-Rp (20 µM) or H89 (10 µM), to block PKA, and the response to PDE-inhibition. Gray shade indicates application of IBMX. N = 4 and 5. Mean ± SEM.

B Quantification of the effect of IBMX on evoked EPSCs. Data points represent averaged responses per cell during a five-minute window after the IBMX application. Unpaired t-test, p = 0.35, N = 4 and 5

C Example recordings from neurons in (A), showing EPSCs before (*black*) and after (*red*) IBMX.

D Time course of EPSC recordings with application of PRKG-inhibitor KT5823 (1 µM, 10 min) indicated by the gray shade. N=4. Mean ± SEM. Example traces to the right.

E Example recordings from neurons in (D), showing EPSCs before (*left*) and after (*right*) KT5823


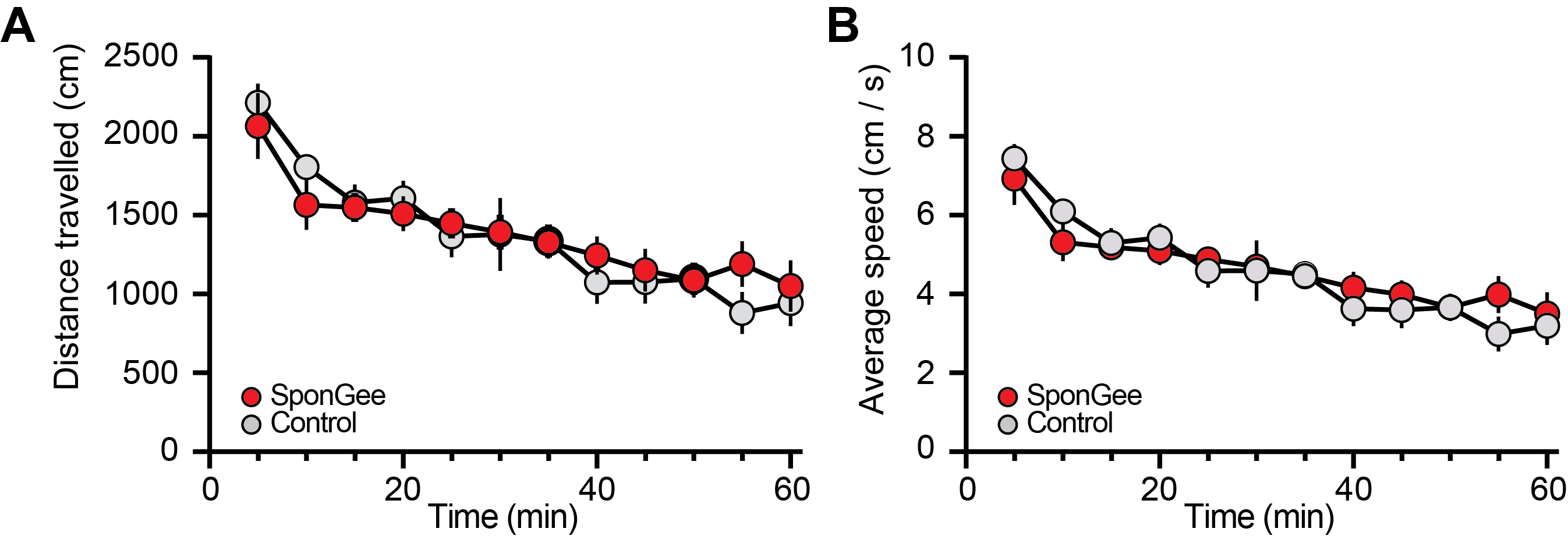


Appendix Fig S7. SponGee expression in M1 cortex does not cause deficits in spontaneous locomotion.

A Mice were tested in an open field arena for 60 minutes. The distance travelled per five-minute bin is shown for both groups. Mean ± SEM. 2-way ANOVA followed by Sidak’s post hoc test.

B The average travelling speed per bin is plotted for the whole duration, for both groups. N = 7 and 8. Mean ± SEM. 2-way ANOVA followed by Sidak’s post hoc test.


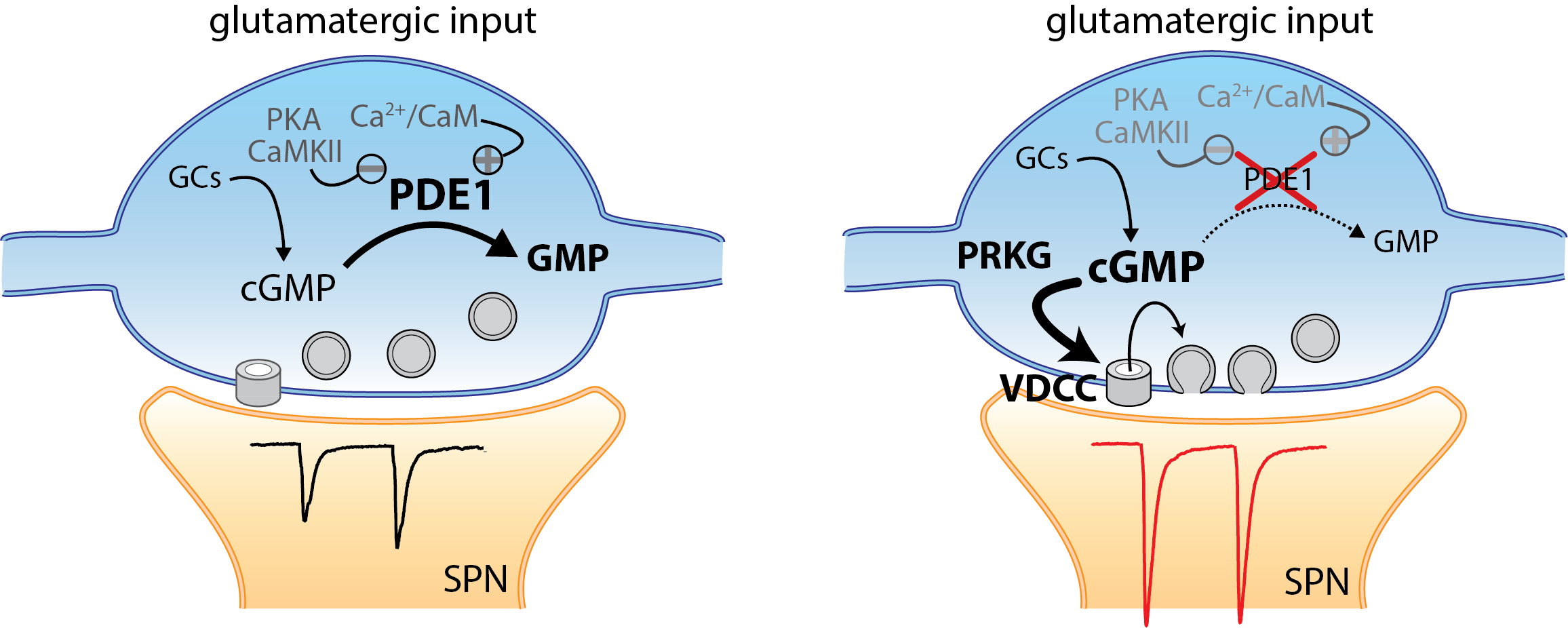


Appendix Fig S8. Graphical summary of the proposed mechanism.

*Left*: a glutamatergic synapse onto a spiny projection neuron (SPN) under control conditions. Constitutively active PDE1 keeps cGMP levels low, PRKG signaling is not engaged, although cGMP is produced by GCs. Under these conditions, activity of PDE1 can be bi-directionally regulated. Calcium and Calmodulin (CaM) increase the enzymatic activity, whereas phosphorylation by either PKA or CaM kinase 2 (CaMKII) decreases it. *Right*: the same synapse under experimental conditions where PDE1 is completely inhibited. Now cGMP activates PRKG, which in turn enhances calcium influx through VDCCs. This leads to increased spontaneous transmitter release, large evoked EPSCs, and reduced PPR.

Appendix Table

Appendix Table S1. Details of statistical tests

| **Figure** | | **Test** | | **P value** | **Comment** |
| --- | --- | --- | --- | --- | --- |
| **1** | **C** | ANOVA | F (4, 25) = 14.79 | <0.0001 |  |
|  |  | Tukey’s multiple comparison test |  | 0.0008 | IBMX vs. Rolipram |
|  |  |  |  | <0.0001 | IBMX vs. Papaverine |
|  |  |  |  | <0.0001 | IBMX vs. BAY 60-7550 |
|  |  |  |  | 0.9542 | IBMX vs. MMPX |
|  |  |  |  | 0.9346 | Rolipram vs. Papaverine |
|  |  |  |  | 0.954 | Rolipram vs. BAY 60-7550 |
|  |  |  |  | 0.0125 | Rolipram vs. MMPX |
|  |  |  |  | >0.9999 | Papaverine vs. BAY 60-7550 |
|  |  |  |  | 0.0012 | Papaverine vs. MMPX |
|  |  |  |  | 0.0015 | BAY 60-7550 vs. MMPX |
|  | **C** | Paired t-test | t = 4.869, df = 6 | 0.0028 | post IBMX vs. baseline |
|  |  | Paired t-test | t = 6.186, df = 4 | 0.0035 | post MMPX vs. baseline |
|  |  | Paired t-test | t = 1.177, df = 4 | 0.3046 | post Rolipram vs. baseline |
|  |  | Paired t-test | t = 0.2524, df =5 | 0.8108 | post Papaverine vs. baseline |
|  |  | Paired t-test | t = 0.7422, df = 5 | 0.4914 | post BAY 60-7550 vs. baseline |
|  |  | Paired t-test | t = 0.1655, df = 3 | 0.8791 | post Vehicle vs. baseline |
|  | **F** | Unpaired t test | t=0.02685, df=15 | 0.9789 | CS vs. TS |
|  | **I** | Unpaired t test | t=1.278, df=9 | 0.2332 | dSPN vs. iSPN |
| **2** | **C** | ANOVA | F (2, 23) = 13.66 | 0.0001 | EPSC |
|  |  | Tukey's multiple comparisons test |  | 0.0221 | 0.4 vs. 2 |
|  |  |  |  | <0.0001 | 0.4 vs. 4 |
|  |  |  |  | 0.0086 | 2 vs. 4 |
|  | **D** | ANOVA | F (2, 23) = 16.79 | <0.0001 | PPR |
|  |  | Tukey's multiple comparisons test |  | 0.0004 | 0.4 vs. 2 |
|  |  |  |  | <0.0001 | 0.4 vs. 4 |
|  |  |  |  | 0.1378 | 2 vs. 4 |
|  | **G** | Paired t-test | t=0.004, df=8 | 0.9971 | mEPSC amplitude |
|  | **I** | Paired t-test | t=8.790, df=8 | <0.0001 | mEPSC frequency |
| **3** | **E** | Paired t-test | t=9.023, df=10 | <0.0001 | Peak jGCaMP7b |
|  | **H** | Paired t-test | t=4.794, df=6 | 0.003 | Peak iGluSnFR |
| **4** | **B** | Unpaired t-test | t=1.055, df=10 | 0.3163 | LY vs. IBMX 🡪 LY |
|  | **E** | Unpaired t-test | t=3.047, df=11 | 0.0111 | BAC vs. IBMX 🡪 BAC |
|  | **H** | Unpaired t-test | t=3.318, df=15 | 0.0047 | LY 🡪 IBMX vs. BAC 🡪 IBMX |
| **5** | **C** | ANOVA | F (4, 23) = 6.316 | 0.0014 |  |
|  |  | Dunnett's multiple comparison test |  | 0.0378 | IBMX vs. IBMX+KT5720 |
|  |  |  |  | 0.9988 | IBMX vs. IBMX + ESI09 |
|  |  |  |  | >0.999 | IBMX vs. IBMX + ZD7288 |
|  |  |  |  | 0.0292 | IBMX vs. IBMX + KT5823 |
|  | **G** | ANOVA | F (2, 14) = 43.8 | <0.0001 |  |
|  |  |  |  | <0.0001 | IBMX vs. IBMX + ODQ |
|  |  |  |  | <0.0001 | IBMX vs. IBMX + PTIO |
|  | **L** | Unpaired t test | t=4.811, df=15 | 0.0002 | SponGee Cortex |
|  | **N** | Unpaired t test | t=5.290, df=10 | 0.0004 | SponGee Thalamus |
| **6** | **B** | Two-way RM ANOVA | F (6, 174) = 22.99 | <0.0001 | LED Power x SponGee |
|  |  |  | F (2.54, 73.53) = 470.5 | <0.0001 | LED Power |
|  |  |  | F (1, 29) = 52.23 | <0.0001 | SponGee |
|  |  |  | F (29, 174) = 3.412 | <0.0001 | Subject |
|  |  | Sidak’s multiple comparison test |  | >0.9999 | 1 % |
|  |  |  |  | 0.1445 | 3 % |
|  |  |  |  | <0.0001 | 5 % |
|  |  |  |  | <0.0001 | 10 % |
|  |  |  |  | <0.0001 | 20 % |
|  |  |  |  | 0.0072 | 30 % |
|  |  |  |  | 0.9928 | 50 % |
|  | **C** | Unpaired t test | t=0.398, df=29 | 0.6935 | Max current |
|  | **D** | Two-way RM ANOVA (mixed effect model) | F (2.31, 66.23) = 1.691 | 0.1878 | Interval |
|  |  |  | F (1, 29) = 1.377 | 0.2502 | SponGee |
|  |  |  | F (3, 86) = 1.581 | 0.2000 | Interval x SponGee |
|  |  | Sidak’s multiple comparison test |  | 0.0326 | 50 ms ISI |
|  |  |  |  | >0.9999 | 100 ms ISI |
|  |  |  |  | 0.9913 | 500 ms ISI |
|  |  |  |  | 0.9844 | 1000 ms ISI |
|  | **E** | Two-way RM ANOVA | F (19, 551) = 6.648 | <0.0001 | Pulse # x SponGee |
|  |  |  | F (3.53, 102.3) = 52.86 | <0.0001 | Pulse # |
|  |  |  | F (1, 29) = 32.05 | <0.0001 | SponGee |
|  |  |  | F (29, 551) = 39.05 | <0.0001 | Subject |
|  |  | Sidak’s multiple comparison test |  | 0.9907 | Pulse #2 |
|  |  |  |  | 0.0098 | Pulse #3 |
|  |  |  |  | 0.0075 | Pulse #4 |
|  |  |  |  | 0.0012 | Pulse #5 |
|  |  |  |  | <0.0001 | Pulse #6 |
|  |  |  |  | 0.0002 | Pulse #7 |
|  |  |  |  | <0.0001 | Pulse #8 |
|  |  |  |  | <0.0001 | Pulse #9 |
|  |  |  |  | <0.0001 | Pulse #10 |
|  |  |  |  | <0.0001 | Pulse #11 |
|  |  |  |  | 0.0002 | Pulse #12 |
|  |  |  |  | <0.0001 | Pulse #13 |
|  |  |  |  | 0.0001 | Pulse #14 |
|  |  |  |  | 0.0002 | Pulse #15 |
|  |  |  |  | 0.0027 | Pulse #16 |
|  |  |  |  | <0.0001 | Pulse #17 |
|  |  |  |  | 0.0004 | Pulse #18 |
|  |  |  |  | 0.0005 | Pulse #19 |
|  |  |  |  | 0.0019 | Pulse #20 |
|  | **F** | Two-way RM ANOVA | F (19, 532) = 5.733 | <0.0001 | Pulse # x SponGee |
|  |  |  | F (4.56, 127.6) = 72.35 | <0.0001 | Pulse # |
|  |  |  | F (1, 28) = 21.48 | <0.0001 | SponGee |
|  |  |  | F (28, 532) = 64.74 | <0.0001 | Subject |
|  |  | Sidak’s multiple comparison test |  | 0.7140 | Pulse #2 |
|  |  |  |  | 0.0965 | Pulse #3 |
|  |  |  |  | 0.0164 | Pulse #4 |
|  |  |  |  | 0.0113 | Pulse #5 |
|  |  |  |  | 0.0010 | Pulse #6 |
|  |  |  |  | 0.0011 | Pulse #7 |
|  |  |  |  | 0.0029 | Pulse #8 |
|  |  |  |  | 0.0003 | Pulse #9 |
|  |  |  |  | 0.0012 | Pulse #10 |
|  |  |  |  | 0.0073 | Pulse #11 |
|  |  |  |  | 0.0072 | Pulse #12 |
|  |  |  |  | 0.0063 | Pulse #13 |
|  |  |  |  | 0.0015 | Pulse #14 |
|  |  |  |  | 0.0191 | Pulse #15 |
|  |  |  |  | 0.0058 | Pulse #16 |
|  |  |  |  | 0.0125 | Pulse #17 |
|  |  |  |  | 0.0108 | Pulse #18 |
|  |  |  |  | 0.0184 | Pulse #19 |
|  |  |  |  | 0.0074 | Pulse #20 |
| **7** | **D** | Two-way RM ANOVA | F (2, 34) = 1.146 | 0.33 | Trial x Group |
|  |  |  | F (2, 34) = 28.35 | <0.0001 | Trial |
|  |  |  | F (1, 17) = 18.14 | 0.0005 | Group |
|  |  |  | F (17, 34) = 0.7782 | 0.7037 | Subject |
|  |  | Sidak’s multiple comparison test |  | 0.7052 | Naïve |
|  |  |  |  | 0.0128 | Early |
|  |  |  |  | 0.216 | Advanced |
|  | **E** | Unpaired t test | t=4.493, df=17 | 0.0003 | Trials over 240 s |
|  | **F** | Unpaired t test | t=2.446, df=17 | 0.0256 | Trials to 300 s |
| **S1** | **B** | Paired t-test | t=3.457, df=4 | 0.0259 | Active Synapse |
| **S2** | **A** | Two-way RM ANOVA | F (19, 171) = 5.789 | <0.0001 | increasing current injection x SPN type |
|  |  |  | F (19, 171) = 30.91 | <0.0001 | increasing current injection |
|  |  |  | F (1, 9) = 7.779 | 0.0211 | SPN type |
|  |  |  | F (9, 171) = 22.31 | <0.0001 | Cells |
|  |  | Sidak's multiple comparisons test |  | 0.0011 < p < 0.0495 |  |
| **S4** | **B** | Paired t-test | t=4.017, df=5 | 0.0102 | ROIs |
| **S5** | **B** | Paired t-test | t=3.440, df=5 | 0.0184 | Cholinergic signaling |
| **S6** | **B** | Unpaired t-test | t=0.997, df=7 | 0.3521 | PKA inhibitor |
| **S7** | **A** | Two-way RM ANOVA | F (11, 143) = 0.9496 | 0.4953 | Time x Group |
|  |  |  | F (5.575, 72.48) = 19.52 | <0.0001 | Time |
|  |  |  | F (1, 13) = 0.02328 | 0.8811 | Group |
|  |  |  | F (13, 143) = 8.202 | <0.0001 | Subject |
|  |  | Sidak's multiple comparisons test |  | 0.83 < p < 0.9999 |  |
|  | **B** | Two-way RM ANOVA | F (11, 143) = 0.9340 | 0.5098 | Time x Group |
|  |  |  | F (5.639, 73.31) = 19.78 | <0.0001 | Time |
|  |  |  | F (1, 13) = 0.03417 | 0.8562 | Group |
|  |  |  | F (13, 143) = 7.008 | <0.0001 | Subject |
|  |  | Sidak's multiple comparisons test |  | 0.83 < p < 0.9999 |  |
