# Supplementary material for: Presynaptic cGMP sets synaptic strength in the striatum and is important for motor learning
Source: EMBO Rep. 2022 Jun 23;23(8):e54361. doi: 10.15252/embr.202154361 (PMC9346481; doi:10.15252/embr.202154361)
Supplement: Supplementary file 2 [file EMBR-23-e54361-s002.pdf]

EMBO reports

Presynaptic cGMP sets synaptic strength in the striatum and is important for motor learning

Tim Fieblinger, Alberto Perez-Alvarez, Paul Lamothe-Molina, Christine Gee, and Thomas Oertner
DOI: 10.15252/embr.202154361

Corresponding author(s): Tim Fieblinger (tim.fieblinger@zmnh.uni-hamburg.de)

Review Timeline:

Submission Date:	19th Nov 21
Editorial Decision:	24th Jan 22
Revision Received:	3rd May 22
Editorial Decision:	26th May 22
Revision Received:	30th May 22
Accepted:	1st Jun 22

Editor: Esther Schnapp

Transaction Report:

(Note: With the exception of the correction of typographical or spelling errors that could be a source of ambiguity, letters and reports are not edited. Depending on transfer agreements, referee reports obtained elsewhere may or may not be included in this compilation. Referee reports are anonymous unless the Referee chooses to sign their reports.)

1st Editorial Decision **24th Jan 2022**

Dear Dr. Fieblinger,

Thank you for the submission of your manuscript to EMBO reports. It was sent to three referees, but so far, we have only received the enclosed comments from two of them. Given that both referees are in fair agreement that you should be given a chance to revise the manuscript, I would like to ask you to begin revising your study along the lines suggested by the referees. Please note that this is a preliminary decision made in the interest of time, and that it is subject to change should the third referee offer very strong and convincing reasons for this. As soon as we will receive the final report on your manuscript, it will be forwarded to you as well.

As you will see, the referees acknowledge that the findings are potentially interesting and novel. However, they also point out that the data should be strengthened and that significant revisions are required before the study can be published here. I think all referee comments are reasonable and should be addressed. If you have any questions or comments, we can also discuss the revisions in a video chat, if you like.

I would thus like to invite you to revise your manuscript with the understanding that the referee concerns must be fully addressed and their suggestions taken on board. Please address all referee concerns in a complete point-by-point response. Acceptance of the manuscript will depend on a positive outcome of a second round of review. It is EMBO reports policy to allow a single round of major revision only and acceptance or rejection of the manuscript will therefore depend on the completeness of your responses included in the next, final version of the manuscript.

We realize that it is difficult to revise to a specific deadline. In the interest of protecting the conceptual advance provided by the work, we recommend a revision within 3 months (26th Apr 2022). Please discuss the revision progress ahead of this time with the editor if you require more time to complete the revisions.

IMPORTANT NOTE: we perform an initial quality control of all revised manuscripts before re-review. Your manuscript will FAIL this control and the handling will be DELAYED if the following APPLIES:
1) A data availability section providing access to data deposited in public databases is missing. If you have not deposited any data, please add a sentence to the data availability section that explains that.
2) Your manuscript contains statistics and error bars based on n=2. Please use scatter blots in these cases. No statistics should be calculated if n=2.

When submitting your revised manuscript, please carefully review the instructions that follow below. Failure to include requested items will delay the evaluation of your revision.

1) a .docx formatted version of the manuscript text (including legends for main figures, EV figures and tables). Please make sure that the changes are highlighted to be clearly visible.

2) individual production quality figure files as .eps, .tif, .jpg (one file per figure). See https://wol-prod-cdn.literatumonline.com/pb-assets/embo-site/EMBOPress_Figure_Guidelines_061115-1561436025777.pdf for more info on how to prepare your figures.

3) We replaced Supplementary Information with Expanded View (EV) Figures and Tables that are collapsible/expandable online. A maximum of 5 EV Figures can be typeset. EV Figures should be cited as 'Figure EV1, Figure EV2" etc... in the text and their respective legends should be included in the main text after the legends of regular figures.

- For the figures that you do NOT wish to display as Expanded View figures, they should be bundled together with their legends in a single PDF file called *Appendix*, which should start with a short Table of Content. Appendix figures should be referred to in the main text as: "Appendix Figure S1, Appendix Figure S2" etc. See detailed instructions regarding expanded view here: <https://www.embopress.org/page/journal/14693178/authorguide#expandedview>

- Additional Tables/Datasets should be labeled and referred to as Table EV1, Dataset EV1, etc. Legends have to be provided in a separate tab in case of .xls files. Alternatively, the legend can be supplied as a separate text file (README) and zipped together with the Table/Dataset file.

4) a .docx formatted letter INCLUDING the reviewers' reports and your detailed point-by-point responses to their comments. As part of the EMBO Press transparent editorial process, the point-by-point response is part of the Review Process File (RPF), which will be published alongside your paper.

5) a complete author checklist, which you can download from our author guidelines <https://www.embopress.org/page/journal/14693178/authorguide>. Please insert information in the checklist that is also reflected in the manuscript. The completed author checklist will also be part of the RPF.

6) Please note that all corresponding authors are required to supply an ORCID ID for their name upon submission of a revised

manuscript (<https://orcid.org/>). Please find instructions on how to link your ORCID ID to your account in our manuscript tracking system in our Author guidelines <https://www.embopress.org/page/journal/14693178/authorguide#authorshipguidelines>

7) Before submitting your revision, primary datasets produced in this study need to be deposited in an appropriate public database (see https://www.embopress.org/page/journal/14693178/authorguide#datadeposition). Please remember to provide a reviewer password if the datasets are not yet public. The accession numbers and database should be listed in a formal "Data Availability" section placed after Materials & Method (see also https://www.embopress.org/page/journal/14693178/authorguide#datadeposition). Please note that the Data Availability Section is restricted to new primary data that are part of this study. * Note - All links should resolve to a page where the data can be accessed. *
If your study has not produced novel datasets, please mention this fact in the Data Availability Section.

8) We would also encourage you to include the source data for figure panels that show essential data. Numerical data should be provided as individual .xls or .csv files (including a tab describing the data). For blots or microscopy, uncropped images should be submitted (using a zip archive if multiple images need to be supplied for one panel). Additional information on source data and instruction on how to label the files are available at <https://www.embopress.org/page/journal/14693178/authorguide#sourcedata>.

9) Our journal also encourages inclusion of *data citations in the reference list* to directly cite datasets that were re-used and obtained from public databases. Data citations in the article text are distinct from normal bibliographical citations and should directly link to the database records from which the data can be accessed. In the main text, data citations are formatted as follows: "Data ref: Smith et al, 2001" or "Data ref: NCBI Sequence Read Archive PRJNA342805, 2017". In the Reference list, data citations must be labeled with "[DATASET]". A data reference must provide the database name, accession number/identifiers and a resolvable link to the landing page from which the data can be accessed at the end of the reference. Further instructions are available at https://www.embopress.org/page/journal/14693178/authorguide#referencesformat

10) Regarding data quantification (see Figure Legends: https://www.embopress.org/page/journal/14693178/authorguide#figureformat)

The following points must be specified in each figure legend:

- the name of the statistical test used to generate error bars and P values,

- the number (n) of independent experiments (please specify technical or biological replicates) underlying each data point,

- the nature of the bars and error bars (s.d., s.e.m.),

- If the data are obtained from n {less than or equal to} 2, use scatter blots showing the individual data points.

Discussion of statistical methodology can be reported in the materials and methods section, but figure legends should contain a basic description of n, P and the test applied.

- Please also include scale bars in all microscopy images.

11) The journal requires a statement specifying whether or not authors have competing interests (defined as all potential or actual interests that could be perceived to influence the presentation or interpretation of an article). In case of competing interests, this must be specified in your disclosure statement. Further information: https://www.embopress.org/competing-interests

We would also welcome the submission of cover suggestions, or motifs to be used by our Graphics Illustrator in designing a cover.

As part of the EMBO publication's Transparent Editorial Process, EMBO reports publishes online a Review Process File (RPF) to accompany accepted manuscripts. This File will be published in conjunction with your paper and will include the referee reports, your point-by-point response and all pertinent correspondence relating to the manuscript.

You are able to opt out of this by letting the editorial office know (emboreports@embo.org). If you do opt out, the Review Process File link will point to the following statement: "No Review Process File is available with this article, as the authors have chosen not to make the review process public in this case."

I look forward to seeing a revised form of your manuscript when it is ready. Please use this link to submit your revision:

https://embor.msubmit.net/cgi-bin/main.plex

Best regards,
Esther

Esther Schnapp, PhD
Senior Editor
EMBO reports

Referee #1:

Fieblinger et al. provide a series of acute slice experiments, which collectively suggest that presynaptic mechanisms involving cGMP, PRKG and presynaptic Ca influx are involved in the potentiation of synaptic transmission between inputs from M1 (primary motor cortex) and PF (thalamic parafascicular nucleus) and medium spiny neurons in dorso-lateral striatum. They further show that expression of a cGMP sponge construct in M1 neurons in vivo leads to an impairment in motor skill learning (rotarod test) in mice.

The discovery that cGMP (and not cAMP) has an important presynaptic role at excitatory input synapses to dorsal striatum is of interest. Furthermore, the experiments are carried out at a high technical standard. On the other hand, the evidence is often based on relatively crude pharmacological evidence (many possible cellular targets) - the sum of the evidence is definitely consistent with the conclusions drawn by the authors, but due to the nature of the evidence one can come up with possible technical limitations for many of the individual conclusions. A main weakness is that the critical in vivo evidence (Fig 6) is based on relatively mild effects (the mice still learn, but not quite as effectively), and the experiment does not conclusively link the behavioural effect to transmission of M1 neurons in dorsolateral striatum (as opposed to other targets of the same M1 neurons expressing the cGMP sponge).
Overall, this is a valuable study, but the key in vivo relevance of the mechanism needs to be more compelling.

Specific points:

1) The conclusion and relevance of the study would be greatly strengthened if the authors could provide additional evidence that presynaptic cGMP has a critical role for in vivo transmission at cortical synapses onto MSNs in dorsolateral striatum and for associated motor learning. That might involve an additional behavioural assay with possibly more dramatic effects of the cGMP sponge and/or more compelling evidence that in these in vivo experiments it is indeed the cortico-striatal (pre)synapse that is involved in the behavioural effects(s). Similar experiments using a cAMP sponge (if available) would also be a good option.

2) The evidence relating the presynaptic PDE effects to Glu transmission (Glu sensor) at postsynaptic striatal MSNs is not as convincing as that involving presynaptic calcium (Fig3).

3) The crucial evidence that the mechanism affects both D1- and D2-MSNs (direct/indirect pathway) in a comparative way would be more convincing if a corresponding Cre mouse would also be used for the D1-MSN evidence (as opposed to an indirect argument based on Cre-negative cells).

4) Why does the PKA inhibitor enhance normalised EPSCs in the experiment shown in Fig5?

5) The sponge experiments are elegant. If there is a similar sponge reagent for cAMP, it would be valuable to add corresponding experiments in order to further corroborate the main conclusions.

6) In the discussion, the authors should add a few lines to elaborate on what advantage(s) might be provided by this particular mechanism specifically at the entry synapses to dorsal striatum.

Referee #2:

The manuscript by Fieblinger et al., describes a set of experiments implicating presynaptic cGMP in the regulation of synaptic strength at glutamatergic synapses innervating the dorsal lateral striatum. The authors used a variety of brain slice electrophysiological and optical techniques, along with pharmacology, genetic manipulations and an assay of motor function. They show that IBMX, a nonselective PDE inhibitor, produces long lasting enhancement of evoked EPSCs; an effect that was reproduced with a selective PDE1 inhibitor, but not several inhibitors of other PDEs. This effect was observed at both corticostriatal and thalamostriatal synapses on dSPNs and iSPNs. Multiple lines of evidence are presented indicating that enhancement of synaptic transmission by PDE inhibition involves a presynaptic calcium-sensitive mechanism leading to increased glutamate release. The authors show that the effect of IBMX is prevented by inhibition of PRKG, but not several cAMP effectors, suggesting that cGMP signaling is responsible for enhanced synaptic transmission. Corroborating this finding,

they show that expression of the genetically-encoded cGMP scavenger, SponGee in either corticostriatal or corticothalamic afferents blocks IBMX effects. Finally, the authors show that expression of SponGee in primary motor cortex disrupts rotarod performance. Collectively, these novel findings demonstrate that cGMP modulates glutamatergic transmission in the DLS, which has important implications regarding synaptic plasticity of striatal circuits involved in action control. The findings are exciting and new, and the experiments are well designed and analyzed appropriately. There are just a few questions and suggestions that the authors should address.

Major critiques:

1. The authors provide evidence that there is not tonic PRKG activation in the absence of PDE inhibition. However, the findings suggest that GC is tonically active in these terminals, as the authors discuss, and thus it would be nice to verify this experimentally if possible. Can this enzyme be selectively inhibited, and if so, would that reduce transmission in the absence of PDE inhibition?

2. Did SponGee expression alter any synaptic transmission parameters in the absence of IBMX?

Minor critiques:

i. Does the time course for the iGluSnFr experiments match the electrophysiology experiments? Can this be presented as a supplement?

ii. Are GC and PRKG expressed in the deep layer cortical and thalamic neurons that innervate striatum? There may be some in situ hybridization or RNAscope data that shows this.

iii. Page 5, 2nd paragraph, the authors should also note that GABABR-mediated inhibition of presynaptic calcium entry was observed in the Kupferschmidt and Lovinger 2015 paper. Thus, this receptor has effects on calcium-dependent as well as calcium-independent mechanisms.

iv. Cartoon in figure 1a implies electrical stimulation selectively activates corticostriatal afferents, however as indicated in the text, thalamocortical afferents are likely activated by electrical stimulation regardless of electrode positioning. The suggestion that corticostriatal afferents are selectively stimulated should be changed.

v. The authors should clearly indicate which control AAV was used for the slice and in vivo SponGee experiments. It would be best to do this in the figures as well as text.

Dear Dr. Fieblinger,

We have now received the final referee report on your manuscript, I paste it below. Please also address these comments and don't hesitate to contact me if you have any questions.

Best regards,
Esther

Esther Schnapp, PhD
Senior Editor
EMBO reports

Referee 3:

This excellent study by Fieblinger et al examines the interesting observation that inhibition of phosphodiesterase 1 significantly enhances corticostriatal and thalamostriatal synaptic transmission. This observation was tested using IBMX inhibition of PDE1, which degrades cAMP and cGMP, to show that under conditions of electrical and optogenetic stimulation of the corticostriatal and thalamostriatal inputs these pathways are substantially enhanced. Furthermore the change appears to be presynaptic as measures of Pr like PPR and mEPSC frequency were increased in IBMX whereas mEPSC amplitude, by contrast was unchanged. Changing extracellular Ca levels suggested that IBMX resulted in increased presynaptic Ca influx. This was confirmed by GCaMP imaging of corticostriatal axon terminals in the striatum. The investigators further showed that PDE inhibition differentially impacts type 2 mGluR vs GABA-B mediated presynaptic changes. Using pharmacology and a genetically encoded cGMP scavenger sponge it was further demonstrated that the effect is most likely mediated by cGMP action on PRKG. These cGMP dependent changes are behaviourally meaningful, as animals expressing SponGee in corticostriatal terminals exhibited impaired motor learning on the rotarod.

1. Overall the phenomenon of PDE-mediated regulation of presynaptic release in the striatum is well characterized and unambiguously demonstrated in this paper. However there are a number of details which would improve the manuscript. The endogenous involvement of this pathway as a plasticity mechanism is difficult to understand, given that, as the authors explain NO levels are naturally high in the striatum and sGC appears to be almost constitutively active under most circumstances. What is the specific evidence for PDE1 in the corticostriatal and thalamostriatal terminals? Is it PDE activity or GC activity or both that are dynamically regulated to control dynamic range of neurotransmission? The mGluR2/3 experiments suggest the possibility that endogenous PDE regulation may be important, but it would be nice do explain, if not experimentally, at least with a clear model how regulation of cGMP levels in this system might occur under natural conditions.

2. Would NOS inhibition or scavenging help implicate NO regulation of sGC in this phenomenon as well? Are there conditions where NO levels might be regulated endogenously?

3. The idea that PRKG may regulate evoked Ca influx to control vesicular release makes sense, though the exact targets by which PRKG works are not discussed. They should be mentioned.

4. It is not necessarily expected that mechanisms that alter evoked Ca levels would also alter miniature EPSC frequency in TTX. The authors should clarify how that mechanism might work, whether these are common or independent phenomena.

5. In Figure 5E SponGee appears to not only render evoked EPSCs insensitive to IBMX, but also significantly enhances their basal size. This is difficult to reconcile with the other data in the paper. Why does scavenging cGMP levels increase rather than reduce synaptic event amplitude? Can the authors provide some explanation? Also is there a cAMP scavenger or degrading construct that could be tested as a control?

6. Given the unexpected change to basal EPSC amplitude in SponGee-expressing axons, can we really attribute the changes in motor learning to a lack of cGMP mediated plasticity or to reduced cGMP levels? Or might it simply be the consequence of whatever baseline potentiation occurs in SponGee expressing axons? This seems to be an important caveat to interpreting the behavioral learning data.

Minor points:
1. What is happening in figure 6C on day 5? It seems the difference between groups collapses transiently.

2. The reference manager puts parentheses around references even when they are part of a parenthetical statement [e.g., (reviewed in (Hardingham et al, 2013))].

We thank the editor and referees for taking the time to review our study and the constructive comments. We hope you will agree that the now resubmitted manuscript has been much improved by the additional experiments that we have performed, and other changes suggested by the referees. Please find our point-by-point reply below.

Point-by-point reply

Referee 1:

Fieblinger et al. provide a series of acute slice experiments, which collectively suggest that presynaptic mechanisms involving cGMP, PRKG and presynaptic Ca influx are involved in the potentiation of synaptic transmission between inputs from M1 (primary motor cortex) and PF (thalamic parafascicular nucleus) and medium spiny neurons in dorso-lateral striatum. They further show that expression of a cGMP sponge construct in M1 neurons in vivo leads to an impairment in motor skill learning (rotarod test) in mice.
The discovery that cGMP (and not cAMP) has an important presynaptic role at excitatory input synapses to dorsal striatum is of interest. Furthermore, the experiments are carried out at a high technical standard. On the other hand, the evidence is often based on relatively crude pharmacological evidence (many possible cellular targets) - the sum of the evidence is definitely consistent with the conclusions drawn by the authors, but due to the nature of the evidence one can come up with possible technical limitations for many of the individual conclusions. A main weakness is that the critical in vivo evidence (Fig 6) is based on relatively mild effects (the mice still learn, but not quite as effectively), and the experiment does not conclusively link the behavioural effect to transmission of M1 neurons in dorsolateral striatum (as opposed to other targets of the same M1 neurons expressing the cGMP sponge).
Overall, this is a valuable study, but the key in vivo relevance of the mechanism needs to be more compelling.

Specific points:
1) The conclusion and relevance of the study would be greatly strengthened if the authors could provide additional evidence that presynaptic cGMP has a critical role for in vivo transmission at cortical synapses onto MSNs in dorsolateral striatum and for associated motor learning. That might involve an additional behavioural assay with possibly more dramatic effects of the cGMP sponge and/or more compelling evidence that in these in vivo experiments it is indeed the cortico-striatal (pre)synapse that is involved in the behavioural effects(s). Similar experiments using a cAMP sponge (if available) would also be a good option.
Unfortunately, the available cAMP-sponge (Lefkimmiatis et al., 2009) only partially attenuates cAMP signaling in cellular systems and does not attenuate, for instance, responses to forskolin, so we did not acquire it and have not used it. We were indeed surprised that merely interfering with cGMP in M1 neurons had a significant effect on rotarod learning as there are other synapses involved in the task including other cortico-striatal (Kupferschmidt and Lovinger, 2017) and cerbello-thalamic synapses (Sakayori et al., 2019), which are not affected by our manipulation. The effect size we observed is however comparable to the effects following ablation of cerebellothalamic tracts in mice (Sakayori et al., 2019), inhibition of striatal mTOR (Bergeron et al., 2014), or observed in Caspr3-deficient (Hirata et al., 2016) or pep-19/pcp4-null mice (Wei et al., 2011), and in premanifest mouse models of Huntington's disease (St-Cyr et al., 2022, Glangetas et al., 2020, Smith et al., 2014). We now explicitly say in the discussion (page 17) that also other cGMP-dependent processes acting in the neurons or at other M1 synapses could contribute to the observed learning deficit.

2) The evidence relating the presynaptic PDE effects to Glu transmission (Glu sensor) at postsynaptic striatal MSNs is not as convincing as that involving presynaptic calcium (Fig3).

Although the postsynaptic neurons express iGluSnFR, it is indeed the presynaptic release of glutamate into the synaptic cleft that is being imaged. We have added this clarification to the manuscript (page 6). Both, the presynaptic calcium and the resulting release of glutamate from the cortical afferents into the synaptic cleft were significantly increased when PDEs were inhibited. Possibly the reviewer finds the glutamate sensor data less convincing due to the sensor's nature and the extremely short time that glutamate remains elevated in the synaptic cleft. Since the kinetics of the glutamate sensor are much faster than the calcium sensor, also the images must be acquired much faster and can appear somewhat 'noisier' compared to those acquired by imaging calcium.

3) The crucial evidence that the mechanism affects both D1- and D2-MSNs (direct/indirect pathway) in a comparative way would be more convincing if a corresponding Cre mouse would also be used for the D1-MSN evidence (as opposed to an indirect argument based on Cre-negative cells).
We consider the DO_DIO "flip fluorescence" labeling approach to be very robust as all the neurons recorded are expressing a fluorescent molecule. Additionally, we never relied solely on the fluorescence but always confirmed that the GFP and tdTomato expressing cells (iSPNs and putative dSPNs, respectively) showed the for SPNs characteristic action potential firing properties and the SPN subpopulation-specific differences in somatic excitability (Appendix Fig S2A-B, Gertler et al., 2008, Fieblinger et al., 2014).
However, to experimentally address the reviewers concern, we now include additional data (Appendix Figure S2C-E) using a different method to strengthen our evidence that PDE inhibition increases synaptic transmission to striatonigral dSPNs (or D1-MSNs). For these experiments, a retro-AAV with the same 'flip fluorescence' construct was injected into the substantia nigra reticulata (SNr) of BAC-adora2a-Cre mice. This way only the dSPNs, which project to the SNr, were retrogradely labeled and expressed tdTomato. The iSPNs (which would be GFP-labeled because of Cre) will not be transduced as they do not project to the SNr. Demonstrating the strength of this strategy there were now no GFP-positive cells in the striatum in this experiment, and IBMX increased EPSCs in the dSPNs.

4) Why does the PKA inhibitor enhance normalised EPSCs in the experiment shown in Fig5?
The PKA inhibitor KT5720 alone did not enhance evoked EPSCs in the striatum, however, in some experiments KT5720 indeed appeared to enhance the effect of IBMX. We don't have an explanation for this effect but it was not consistent and seemed unique to the compound KT5720, as similar observations were not made with the other PKA inhibitors (see Appendix Figure S6). We now state this in the results (page 10).

5) The sponge experiments are elegant. If there is a similar sponge reagent for cAMP, it would be valuable to add corresponding experiments in order to further corroborate the main conclusions.
Please see the answer to point 1) above.

6) In the discussion, the authors should add a few lines to elaborate on what advantage(s) might be provided by this particular mechanism specifically at the entry synapses to dorsal striatum.
We thank for the advice. In combination with our reply to reviewer 3 on the question of dynamic regulators, we have now addressed this point in the discussion on page 15.

Referee 2:

The manuscript by Fieblinger et al., describes a set of experiments implicating presynaptic cGMP in the regulation of synaptic strength at glutamatergic synapses innervating the dorsal lateral striatum. The authors used a variety of brain slice electrophysiological and optical techniques, along with pharmacology, genetic manipulations and an assay of motor function. They show that IBMX, a nonselective PDE inhibitor, produces long lasting enhancement of evoked EPSCs; an effect that was reproduced with a selective PDE1 inhibitor, but not several inhibitors of other PDEs. This effect was observed at both corticostriatal and thalamostriatal synapses on dSPNs and iSPNs. Multiple lines of evidence are presented indicating that enhancement of synaptic transmission by PDE inhibition involves a presynaptic calcium-sensitive mechanism leading to increased glutamate release. The authors show that the effect of IBMX is prevented by inhibition of PRKG, but not several cAMP effectors, suggesting that cGMP signaling is responsible for enhanced synaptic transmission. Corroborating this finding, they show that expression of the genetically-encoded cGMP scavenger, SponGee in either corticostriatal or corticothalamic afferents blocks IBMX effects. Finally, the authors show that expression of SponGee in primary motor cortex disrupts rotarod performance. Collectively, these novel findings demonstrate that cGMP modulates glutamatergic transmission in the DLS, which has important implications regarding synaptic plasticity of striatal circuits involved in action control. The findings are exciting and new, and the experiments are well designed and analyzed appropriately. There are just a few questions and suggestions that the authors should address.

Major critiques:

1. The authors provide evidence that there is not tonic PRKG activation in the absence of PDE inhibition. However, the findings suggest that GC is tonically active in these terminals, as the authors discuss, and thus it would be nice to verify this experimentally if possible. Can this enzyme be selectively inhibited, and if so, would that reduce transmission in the absence of PDE inhibition?
We have now used the NO-GC inhibitor ODQ to dampen cGMP production in the slices. In line with our working hypothesis, ODQ by itself did not affect EPSCs as the PDEs maintain resting cGMP concentration below the threshold for causing changes to presynaptic glutamate release i.e. the PDEs out-compete the GCs. However, ODQ strongly attenuated the boosting effect of IBMX suggesting that NO-GCs contribute to cGMP production in the presynaptic neurons. These new data are incorporated in Fig 5.

2. Did SponGee expression alter any synaptic transmission parameters in the absence of IBMX?
We have performed additional experiments which are found in the new Fig 6. In brief, SponGee does not seem to alter EPSC amplitude but it drastically changed short-term plasticity, as measured by paired-pulse ratios and responses to 10 and 20 Hz trains.

Minor critiques:

i. Does the time course for the iGluSnFr experiments match the electrophysiology experiments? Can this be presented as a supplement?
The time course of the experiments was indeed very similar, although we performed the electrophysiological and imaging experiments on different setups, with not identical perfusion systems and flow rates. A time course example of the imaging experiments, compared to the electrophysiological recordings, is now added in Appendix Fig S4C.

ii. Are GC and PRKG expressed in the deep layer cortical and thalamic neurons that innervate striatum? There may be some in situ hybridization or RNAscope data that shows this.
We have added references to the discussion that have demonstrated expression of NO-GC, PRKG and PDE1 in the deep layers of the cortex and wide thalamic areas, high likely encompassing the projections to the striatum (page 16).

iii. Page 5, 2nd paragraph, the authors should also note that GABABR-mediated inhibition of presynaptic calcium entry was observed in the Kupferschmidt and Lovinger 2015 paper. Thus, this receptor has effects on calcium-dependent as well as calcium-independent mechanisms.
Good point, we now mention this.

iv. Cartoon in figure 1a implies electrical stimulation selectively activates corticostriatal afferents, however as indicated in the text, thalamocortical afferents are likely activated by electrical stimulation regardless of electrode positioning. The suggestion that corticostriatal afferents are selectively stimulated should be changed.
We have modified the cartoon to better show both inputs.

v. The authors should clearly indicate which control AAV was used for the slice and in vivo SponGee experiments. It would be best to do this in the figures as well as text.
In addition to the methods section, we have now added this information to the figure legend and the main text (page 12).

Referee 3:

This excellent study by Fieblinger et al examines the interesting observation that inhibition of phosphodiesterase 1 significantly enhances corticostriatal and thalamostriatal synaptic transmission. This observation was tested using IBMX inhibition of PDE1, which degrades cAMP and cGMP, to show that under conditions of electrical and optogenetic stimulation of the corticostriatal and thalamostriatal inputs these pathways are substantially enhanced. Furthermore the change appears to be presynaptic as measures of Pr like PPR and mEPSC frequency were increased in IBMX whereas mEPSC amplitude, by contrast was unchanged. Changing extracellular Ca levels suggested that IBMX resulted in increased presynaptic Ca influx. This was confirmed by GCaMP imaging of corticostriatal axon terminals in the striatum. The investigators further showed that PDE inhibition differentially impacts type 2 mGluR vs GABA-B mediated presynaptic changes. Using pharmacology and a genetically encoded cGMP scavenger sponge it was further demonstrated that the effect is most likely mediated by cGMP action on PRKG. These cGMP dependent changes are behaviourally meaningful, as animals expressing SponGee in corticostriatal terminals exhibited impaired motor learning on the rotarod.

1. Overall the phenomenon of PDE-mediated regulation of presynaptic release in the striatum is well characterized and unambiguously demonstrated in this paper. However there are a number of details which would improve the manuscript. The endogenous involvement of this pathway as a plasticity mechanism is difficult to understand, given that, as the authors explain NO levels are naturally high in the striatum and sGC appears to be almost constitutively active under most circumstances. What is the specific evidence for PDE1 in the corticostriatal and thalamostriatal terminals? Is it PDE activity or GC activity or both that are dynamically regulated to control dynamic range of neurotransmission? The mGluR2/3 experiments suggest the possibility that endogenous PDE regulation may be important, but it would be nice do explain, if not experimentally, at least with a clear model how regulation of cGMP levels in this system might occur under natural conditions.

We have added references to the discussion demonstrating the expression of PDE1, NO-GC and PRKG in cortical and thalamic neurons (information also requested by reviewer 2). Regarding the questions of dynamic control and natural regulation, we currently favor the hypothesis that the GCs are constitutively active (although this does not preclude some activity-dependent modification) and that the main bi-directional modulation is via the regulation of PDE1 activity. We have added this information to the discussion (page 15) and also adapted the working model cartoon (Appendix Fig. S8) accordingly.

2. Would NOS inhibition or scavenging help implicate NO regulation of sGC in this phenomenon as well? Are there conditions where NO levels might be regulated endogenously?

We have added new data showing that NO scavenging with carboxyl-PTIO dramatically reduced the EPSC enhancement by IBMX. These data are in Figure 5.

Indeed, activity of several NOS isoforms has been found to be Ca^{2+}-dependent and can furthermore be modulated by phosphorylation through e.g. CaMKII. We added this notion in the discussion (page 15).

3. The idea that PRKG may regulate evoked Ca influx to control vesicular release makes sense, though the exact targets by which PRKG works are not discussed. They should be mentioned.

Thank you for pointing out this gap. We now reference the known presynaptic modulation of VGCCs and the ready-releasable pool of synaptic vesicles by PRKG downstream of NO (page 10).

4. It is not necessarily expected that mechanisms that alter evoked Ca levels would also alter miniature EPSC frequency in TTX. The authors should clarify how that mechanism might work, whether these are common or independent phenomena.

It is true that altered evoked Ca^{2+} levels not necessarily predict enhanced spontaneous release (i.e. an increase in mEPSC frequency) as well, and we did not perform additional experiments to unravel the mechanisms underlying our mEPSC effects. However, there are several studies that show that altering presynaptic Ca^{2+} can substantially influence mEPSCs. For example, release of Ca^{2+} from intracellular stores, or stimulating Ca^{2+}-induced-Ca^{2}-release (CICR), changes the mEPSC frequency in pyramidal neurons (Simkus and Stricker, 2002; Sharma and Vijayaraghavan. 2003). Furthermore, BDNF increases hippocampal mEPSC frequency depending on both Ca^{2+}-influx and release from stores (Amaral and Pozzo-Miller, 2012) and chelating Ca^{2+} with e.g. BAPTA-AM reduces mEPSC frequency by altering presynaptic VDCC mobility (Schneider et al., 2015). Miniature EPSCs recorded in cerebellar purkinje cells are similarly sensitive to the $[Ca^{2+}]_e$ and BAPTA-AM loading (Yamasaki et al., 2006). Importantly, in cultured hippocampal neurons, a glutamate-induced increase of mEPSC frequency has been show to occur with a transient increase presynaptic cGMP, PRKG activation and increased synaptophysin puncta (Wang et al., 2005). This is in line with previous findings that in hippocampal cultures, application of a cGMP analog can increase mEPSC frequency through a presynaptic mechanism (Arancio et al., 1995) and provides a potential link to our study. We mention this now in the discussion (page 17).

5. In Figure 5E SponGee appears to not only render evoked EPSCs insensitive to IBMX, but also significantly enhances their basal size. This is difficult to reconcile with the other data in the paper. Why does scavenging cGMP levels increase rather than reduce synaptic event amplitude? Can the authors provide some explanation? Also is there a cAMP scavenger or degrading construct that could be tested as a control?
Unfortunately, there is not a sufficiently effective cAMP scavenger, i.e. one that can decrease forskolin-induced cAMP (see also our replies to reviewer 1). We have added in-depth characterization of the effects of SponGee. Please see new Figure 6, and the responses to the other referees. There is no effect of SponGee on EPSC amplitude per se but it does affect short-term plasticity (PPR and trains).

6. Given the unexpected change to basal EPSC amplitude in SponGee-expressing axons, can we really attribute the changes in motor learning to a lack of cGMP mediated plasticity or to reduced cGMP levels? Or might it simply be the consequence of whatever baseline potentiation occurs in SponGee expressing axons? This seems to be an important caveat to interpreting the behavioral learning data.
While we didn't observe a difference in EPSC amplitude, we did observe that SponGee-expressing axons showed different short-term plasticity in addition to the altered responses to IBMX. It therefore is correct that it might not only be the cGMP-dependent enhancement of EPSCs revealed by IBMX that contributes to motor learning but also the changes in short-term plasticity. We now mention this in the discussion (page 17).

Minor points:
1. What is happening in figure 6C on day 5? It seems the difference between groups collapses transiently.
We unfortunately don't have an explanation for this. The experiments were performed blind and the mice were housed in mixed cages, to which they were returned between the different trials. For the specific trial 5 on day 1, we weren't aware of any disturbances, e.g. in the building or similar, although this cannot be ruled out. Importantly, in the trial immediately following this one, and also for the remaining trials of the day, the mice behaved consistently.

2. The reference manager puts parentheses around references even when they are part of a parenthetical statement [e.g., (reviewed in (Hardingham et al, 2013))].

We have fixed this.

1st Revision - Editorial Decision 26th May 2022

Dear Dr. Fieblinger,

Thank you for the submission of your revised manuscript. We have now received the enclosed reports from the referees that were asked to assess it, and I am happy to say that both support its publication now. Only a few editorial requests still need to be addressed before we can proceed with the acceptance of your manuscript:

- Please correct the conflict of interest subheading to "Disclosure and Competing Interest Statement".

- The callouts for Figure 3I, Appendix Fig S1A, S2D&E, S3A&B, S5A-C, S7A&B are missing, please add to the manuscript text.

- There is a callout to Appendix Fig S9, but there is no such figure. Please correct.

- There is a callout to Supplementary Table 1 which needs removing/correcting.

- The APPENDIX file has a table of content, but page numbers are missing. Please add.

- Please remove the figures from the manuscript file and move the legends to after the Reference section.

- Remove the individually uploaded appendix figures.

EMBO press papers are accompanied online by A) a short (1-2 sentences) summary of the findings and their significance, B) 2-3 bullet points highlighting key results and C) a synopsis image that is exactly 550 pixels wide and 200-600 pixels high (the height is variable). You can either show a model or key data in the synopsis image. Please note that text needs to be readable at the final size. Please send us this information along with the final manuscript.

I look forward to seeing a new revised version of your manuscript as soon as possible. Please use this link to submit your revision: https://embor.msubmit.net/cgi-bin/main.plex

Best regards,
Esther

Esther Schnapp, PhD
Senior Editor
EMBO reports

Referee #1:

The authors provide a somewhat minimalistic set of effective responses to the points raised by Rev1 and Rev3.
However, overall, the manuscript is sufficiently strengthened and the findings of sufficient interest to the field to warrant publication as is.
It would have been nice if the authors had provided more compelling evidence for the role of the cGMP mechanism in vivo, but this will have to be addressed in future studies.

Referee #2:

The authors have adequately addressed all reviewers' comments. This is an interesting and timely report.

The authors have addressed all minor editorial requests

2nd Revision - Editorial Decision 1st Jun 2022

Dr. Tim Fieblinger
Center for Molecular Neurobiology, University Medical Center Hamburg-Eppendorf
Institute for Synaptic Physiology
Falkenried 94
Hamburg 20251
Germany

Dear Dr. Fieblinger,

I am very pleased to accept your manuscript for publication in the next available issue of EMBO reports. Thank you for your contribution to our journal.

At the end of this email I include important information about how to proceed. Please ensure that you take the time to read the information and complete and return the necessary forms to allow us to publish your manuscript as quickly as possible.

Please note that under the DEAL agreement of German scientific institutions with our publisher Wiley, you could be eligible for publication of your article in the open access format in a way that is free of charge for the authors. Please contact either the administration at your institution or our publishers at Wiley (emboreports@wiley.com) for further questions.

As part of the EMBO publication's Transparent Editorial Process, EMBO reports publishes online a Review Process File to accompany accepted manuscripts. As you are aware, this File will be published in conjunction with your paper and will include the referee reports, your point-by-point response and all pertinent correspondence relating to the manuscript.

If you do NOT want this File to be published, please inform the editorial office within 2 days, if you have not done so already, otherwise the File will be published by default [contact: emboreports@embo.org]. If you do opt out, the Review Process File link will point to the following statement: "No Review Process File is available with this article, as the authors have chosen not to make the review process public in this case." Please note that the author checklist will still be published even if you opt out of the transparent process.

Thank you again for your contribution to EMBO reports and congratulations on a successful publication. Please consider us again in the future for your most exciting work.

Best regards,
Esther

Esther Schnapp, PhD
Senior Editor
EMBO reports

****

THINGS TO DO NOW:

Please note that you will be contacted by Wiley Author Services to complete licensing and payment information. The required 'Page Charges Authorization Form' is available here: https://www.embopress.org/pb-assets/embo-site/er_apc.pdf

You will receive proofs by e-mail approximately 2-3 weeks after all relevant files have been sent to our Production Office; you should return your corrections within 2 days of receiving the proofs.

Please inform us if there is likely to be any difficulty in reaching you at the above address at that time. Failure to meet our deadlines may result in a delay of publication, or publication without your corrections.

All further communications concerning your paper should quote reference number EMBOR-2021-54361V3 and be addressed to emboreports@wiley.com.

Should you be planning a Press Release on your article, please get in contact with emboreports@wiley.com as early as possible, in order to coordinate publication and release dates.

EMBO Press Author Checklist

Corresponding Author Name: Tim Fieblinger	
Journal Submitted to: EMBO Reports	
Manuscript Number: EMBOR-2021-54361	

USEFUL LINKS FOR COMPLETING THIS FORM
The EMBO Journal - Author Guidelines
EMBO Reports - Author Guidelines
Molecular Systems Biology - Author Guidelines
EMBO Molecular Medicine - Author Guidelines

Reporting Checklist for Life Science Articles (updated January 2022)
This checklist is adapted from Materials Design Analysis Reporting (MDAR) Checklist for Authors. MDAR establishes a minimum set of requirements in transparent reporting in the life sciences (see Statement of Task: 10.31222/osf.io/9sm4x). Please follow the journal's guidelines in preparing your manuscript.
Please note that a copy of this checklist will be published alongside your article.

Abridged guidelines for figures
1. Data
The data shown in figures should satisfy the following conditions:
- the data were obtained and processed according to the field's best practice and are presented to reflect the results of the experiments in an accurate and unbiased manner.
- ideally, figure panels should include only measurements that are directly comparable to each other and obtained with the same assay.
- plots include clearly labeled error bars for independent experiments and sample sizes. Unless justified, error bars should not be shown for technical replicates.
- if n<5, the individual data points from each experiment should be plotted. Any statistical test employed should be justified.
- Source Data should be included to report the data underlying figures according to the guidelines set out in the authorship guidelines on Data Presentation.

2. Captions
Each figure caption should contain the following information, for each panel where they are relevant:
- a specification of the experimental system investigated (eg cell line, species name).
- the assay(s) and method(s) used to carry out the reported observations and measurements.
- an explicit mention of the biological and chemical entity(ies) that are being measured.
- an explicit mention of the biological and chemical entity(ies) that are altered/varied/perturbed in a controlled manner.
- the exact sample size (n) for each experimental group/condition, given as a number, not a range;
- a description of the sample collection allowing the reader to understand whether the samples represent technical or biological replicates (including how many animals, litters, cultures, etc.).
- a statement of how many times the experiment shown was independently replicated in the laboratory.
- definitions of statistical methods and measures:
  - common tests, such as t-test (please specify whether paired vs. unpaired), simple χ2 tests, Wilcoxon and Mann-Whitney tests, can be unambiguously identified by name only, but more complex techniques should be described in the methods section;
  - are tests one-sided or two-sided?
  - are there adjustments for multiple comparisons?
  - exact statistical test results, e.g., P values = x but not P values < x;
  - definition of 'center values' as median or average;
  - definition of error bars as s.d. or s.e.m.

Please complete ALL of the questions below.
Select "Not Applicable" only when the requested information is not relevant for your study.

Materials

Newly Created Materials	Information included in the manuscript?	In which section is the information available? (Reagents and Tools Table, Materials and Methods, Figures, Data Availability Section)
New materials and reagents need to be available; do any restrictions apply?	Not Applicable	

Antibodies	Information included in the manuscript?	In which section is the information available? (Reagents and Tools Table, Materials and Methods, Figures, Data Availability Section)
For **antibodies** provide the following information:<br>- Commercial antibodies: RRID (if possible) or supplier name, catalogue number and or/clone number<br>- Non-commercial: RRID or citation	Not Applicable	

DNA and RNA sequences	Information included in the manuscript?	In which section is the information available? (Reagents and Tools Table, Materials and Methods, Figures, Data Availability Section)
**Short novel DNA or RNA including primers, probes**: provide the sequences.	Not Applicable	

Cell materials	Information included in the manuscript?	In which section is the information available? (Reagents and Tools Table, Materials and Methods, Figures, Data Availability Section)
**Cell lines:** Provide species information, strain. Provide accession number in repository **OR** supplier name, catalog number, clone number, and/**OR** RRID.	Not Applicable	
**Primary cultures:** Provide species, strain, sex of origin, genetic modification status.	Not Applicable	
Report if the cell lines were recently **authenticated** (e.g., by STR profiling) and tested for mycoplasma contamination.	Not Applicable	

Experimental animals	Information included in the manuscript?	In which section is the information available? (Reagents and Tools Table, Materials and Methods, Figures, Data Availability Section)
**Laboratory animals or Model organisms:** Provide species, strain, sex, age, genetic modification status. Provide accession number in repository **OR** supplier name, catalog number, clone number, **OR** RRID.	Yes	Material and Methods
**Animal observed in or captured from the field:** Provide species, sex, and age where possible.	Not Applicable	
Please detail **housing and husbandry conditions**.	Yes	Material and Methods

Plants and microbes	Information included in the manuscript?	In which section is the information available? (Reagents and Tools Table, Materials and Methods, Figures, Data Availability Section)
**Plants:** provide species and strain, ecotype and cultivar where relevant, unique accession number if available, and source (including location for collected wild specimens).	Not Applicable	
**Microbes:** provide species and strain, unique accession number if available, and source.	Not Applicable	

Human research participants	Information included in the manuscript?	In which section is the information available? (Reagents and Tools Table, Materials and Methods, Figures, Data Availability Section)
If collected and within the bounds of privacy constraints report on age, sex and gender or ethnicity for all study participants.	Not Applicable	

Core facilities	Information included in the manuscript?	In which section is the information available? (Reagents and Tools Table, Materials and Methods, Figures, Data Availability Section)
If your work benefited from core facilities, was their service mentioned in the acknowledgments section?	Not Applicable	

Design

Study protocol	Information included in the manuscript?	In which section is the information available? (Reagents and Tools Table, Materials and Methods, Figures, Data Availability Section)
If study protocol has been **pre-registered, provide DOI in the manuscript**. For clinical trials, provide the trial registration number **OR** cite DOI.	Not Applicable	
Report the **clinical trial registration number** (at ClinicalTrials.gov or equivalent), where applicable.	Not Applicable	

Laboratory protocol	Information included in the manuscript?	In which section is the information available? (Reagents and Tools Table, Materials and Methods, Figures, Data Availability Section)
Provide DOI OR other citation details if **external detailed step-by-step protocols** are available.	Not Applicable	

Experimental study design and statistics	Information included in the manuscript?	In which section is the information available? (Reagents and Tools Table, Materials and Methods, Figures, Data Availability Section)
Include a statement about **sample size** estimate even if no statistical methods were used.	Yes	Material and Methods
Were any steps taken to minimize the effects of subjective bias when allocating animals/samples to treatment (e.g. **randomization procedure**)? If yes, have they been described?	Yes	Material and Methods
Include a statement about **blinding** even if no blinding was done.	Yes	Material and Methods
Describe **inclusion/exclusion criteria** if samples or animals were excluded from the analysis. Were the criteria pre-established?<br><br>If sample or data points were omitted from analysis, report if this was due to attrition or intentional exclusion and provide justification.	Yes	Material and Methods
For every figure, are **statistical tests** justified as appropriate? Do the data meet the assumptions of the tests (e.g., normal distribution)? Describe any methods used to assess it. Is there an estimate of variation within each group of data? Is the variance similar between the groups that are being statistically compared?	Yes	Material and Methods

Sample definition and in-laboratory replication	Information included in the manuscript?	In which section is the information available? (Reagents and Tools Table, Materials and Methods, Figures, Data Availability Section)
In the figure legends: state number of times the experiment was **replicated** in laboratory.	Yes	Material and Methods
In the figure legends: define whether data describe **technical or biological replicates**.	Not Applicable	

Ethics

Ethics	Information included in the manuscript?	In which section is the information available? (Reagents and Tools Table, Materials and Methods, Figures, Data Availability Section)
Studies involving **human participants**: State details of **authority granting ethics approval** (IRB or equivalent committee(s), provide reference number for approval.	Not Applicable	
Studies involving **human participants**: Include a statement confirming that **informed consent** was obtained from all subjects and that the experiments conformed to the principles set out in the WMA Declaration of Helsinki and the Department of Health and Human Services Belmont Report.	Not Applicable	
Studies involving **human participants:** For publication of **patient photos**, include a statement confirming that consent to publish was obtained.	Not Applicable	
Studies involving experimental **animals**: State details of **authority granting ethics approval** (IRB or equivalent committee(s), provide reference number for approval. Include a statement of compliance with ethical regulations.	Yes	Material and Methods
Studies involving **specimen and field samples:** State if relevant **permits** obtained, provide details of authority approving study; if none were required, explain why.	Not Applicable	

Dual Use Research of Concern (DURC)	Information included in the manuscript?	In which section is the information available? (Reagents and Tools Table, Materials and Methods, Figures, Data Availability Section)
Could your study fall under dual use research restrictions? Please check biosecurity documents and list of **select agents and toxins** (CDC): https://www.selectagents.gov/sat/list.htm	Not Applicable	
If you used a select agent, is the security level of the lab appropriate and reported in the manuscript?	Not Applicable	
If a study is subject to dual use research of concern regulations, is the name of the **authority granting approval and reference number** for the regulatory approval provided in the manuscript?	Not Applicable	

Reporting

The MDAR framework recommends adoption of discipline-specific guidelines, established and endorsed through community initiatives. Journals have their own policy about requiring specific guidelines and recommendations to complement MDAR.

Adherence to community standards	Information included in the manuscript?	In which section is the information available? (Reagents and Tools Table, Materials and Methods, Figures, Data Availability Section)
State if relevant guidelines or checklists (e.g., **ICMJE, MIBBI, ARRIVE, PRISMA**) have been followed or provided.	Yes	Material and Methods
For **tumor marker prognostic studies,** we recommend that you follow the **REMARK** reporting guidelines (see link list at top right). See author guidelines, under 'Reporting Guidelines'. Please confirm you have followed these guidelines.	Not Applicable	
For **phase II and III randomized controlled trials**, please refer to the **CONSORT** flow diagram (see link list at top right) and submit the CONSORT checklist (see link list at top right) with your submission. See author guidelines, under 'Reporting Guidelines'. Please confirm you have submitted this list.	Not Applicable	

Data Availability

Data availability	Information included in the manuscript?	In which section is the information available? (Reagents and Tools Table, Materials and Methods, Figures, Data Availability Section)
Have **primary datasets** been deposited according to the journal's guidelines (see 'Data Deposition' section) and the respective accession numbers provided in the Data Availability Section?	Not Applicable	
Were **human clinical and genomic datasets** deposited in a public access-controlled repository in accordance to ethical obligations to the patients and to the applicable consent agreement?	Not Applicable	
Are **computational models** that are central and integral to a study available without restrictions in a machine-readable form? Were the relevant accession numbers or links provided?	Not Applicable	
If publicly available data were reused, provide the respective **data citations in the reference list**.	Not Applicable	
